# Viral vaccines promote endoplasmic reticulum stress-induced unfolding protein response in teleost erythrocytes

**DOI:** 10.1016/j.ejcb.2025.151490

**Published:** 2025-06

**Authors:** Maria Salvador-Mira, Paula Gimenez-Moya, Alba Manso-Aznar, Ester Sánchez-Córdoba, Manuel A. Sevilla-Diez, Veronica Chico, Ivan Nombela, Sara Puente-Marin, Nerea Roher, Luis Perez, Tanja Dučić, Núria Benseny-Cases, Ana Joaquina Perez-Berna, Maria del Mar Ortega-Villaizan

**Affiliations:** a Instituto de Investigación, Desarrollo e Innovación en Biotecnología Sanitaria de Elche (IDiBE), Universidad Miguel Hernández (IDiBE-UMH), Elche, Spain; b Institute of Biotechnology and Biomedicine (IBB) & Department of Cellular Biology, Physiology and Immunology, Universitat Autònoma de Barcelona, Barcelona, Spain; c ALBA Synchrotron Light Source, Cerdanyola del Vallès, Barcelona, Spain; d Department of Biochemistry and Molecular Biology, Universitat Autònoma de Barcelona, Barcelona, Spain

**Keywords:** red blood cells, vaccine, endoplasmic reticulum stress, antiviral immune response, cryo-SXT, SR-µFTIR

## Abstract

Most available evidence points to a proviral role for endoplasmic reticulum (ER) stress, as many viruses exploit it to promote viral replication. In contrast, few studies have linked ER stress to the antiviral immune response, and even fewer to the vaccine-induced immune response. In this work, we demonstrated that ER stress is a key molecular link in the immune response of teleost erythrocytes or red blood cells (RBCs) under vaccine stimulation. Moreover, the unfolded protein response (UPR^ER^) triggered by ER stress may work together with autophagy and related cellular mechanisms as part of a coordinated immune response in RBCs. We unveiled biochemical changes in the lipid-protein profile of vaccine-treated RBCs by synchrotron radiation-based Fourier transform infrared microspectroscopy (SR-µFTIR) associated with the modulation of ER expansion, increased mitochondrial number, and vesicular structures detected by soft X-ray cryotomography (cryo-SXT). We found a positive correlation between both morphological and biochemical changes and the expression of genes related to UPR^ER^, autophagy, mitochondrial stress, vesicle trafficking, and extracellular vesicle release. These processes in RBCs are ideal cellular targets for the development of more specific prophylactic tools with greater immunogenic capacity than currently available options.

## Highlights

• First intracellular interrogation of RBCs using correlative synchrotron radiation techniques.
• Viral vaccines have impacts on the cellular architecture of rainbow trout RBCs.
• Changes in the lipid-protein profile of RBCs are dominant in response to viral vaccines.
• Viral vaccines reshape ER stress via UPR^ER^ activation in rainbow trout RBCs.
• UPR^ER^ may be novel RBC target for prophylactic development.

## Graphical Abstract

**Figure:**
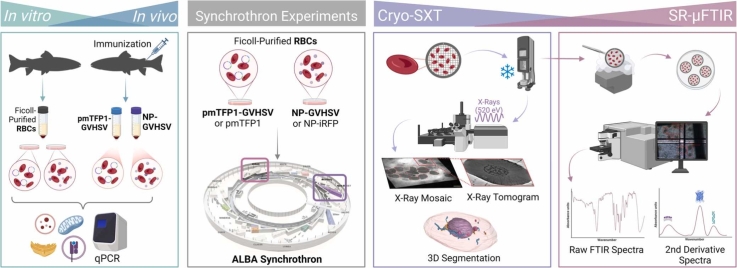

## 1 Introduction

In addition to the production of cytokine-like factors, antigen presentation, and regulation of viral response genes reported for nucleated erythrocytes or red blood cells (RBCs) (Dahle et al., 2015, Jiang et al., 2010, Morera et al., 2011, Nombela et al., 2017, Nombela et al., 2018, Nombela et al., 2019, Passantino et al., 2002, Passantino et al., 2007, Workenhe et al., 2008), autophagy, a degradation pathway that can be cytoprotective under stress conditions, occurs in teleost RBCs in response to viral hemorrhagic septicemia virus (VHSV) as an antiviral and antigen processing mechanism (Nombela et al., 2019, Pereiro et al., 2017).

Notably, autophagy can be stimulated by endoplasmic reticulum (ER) stress within the cell (Deegan et al., 2013, Kabir et al., 2018). The ER acts as a central organelle, forming specialized sites that interconnect the ER with almost all organelles, coordinating stress responses (Borgese et al., 2006, Csordas et al., 2006, Csordas et al., 2018, Wu et al., 2018). Moreover, the ER is responsible for a variety of essential cellular functions, including the correct folding of proteins (Perkins and Allan, 2021, Schwarz and Blower, 2015). Normal function of the ER may be compromised by cellular stress caused by physiological and pathological conditions such as changes in pH and temperature, viral and bacterial attacks, all of which can lead to the accumulation of misfolded proteins (Smith, 2018).

To safeguard cellular homeostasis, ER stress triggers complex signal transduction pathways, collectively known as the unfolded protein response (UPR^ER^). This process is controlled by at least 3 transmembrane proteins acting as stress sensors (Hetz, 2012, Oakes and Papa, 2015, Smith, 2018): double-stranded RNA-activated ER protein kinase (PERK), inositol-requiring enzyme 1 alpha (IRE1α), and activating transcription factor 6 (ATF6). In their inactive states, these transmembrane proteins associate with immunoglobulin-binding protein (BiP; also known as 78 kDa glucose-regulated protein [GRP78]), a chaperone that keeps them dormant when bound. Under conditions of cellular stress, misfolded proteins hijack GRP78, dissociating it from PERK, IRE1α, and ATF6. This process activates pro-survival pathways that restore cellular homeostasis. Viral infections exploit and induce ER stress. For example, protein synthesis is increased to form new virions during the infection cycle. However, the UPR^ER^ appears to limit the replication of certain viruses, which indicates a possible stress-mediated antiviral response of the host cell via the ER (Salvador-Mira et al., 2024, Smith, 2018).

The impact of UPR^ER^ signaling pathways on cellular machinery appears to be an important component of the RBC immune responses against invading pathogens. While the mechanisms of virus-RBC interaction, replication, and invasion have been extensively studied, the influence of RBC organelle alterations on immune function is less well understood. For this reason, we aimed to describe cellular mechanisms associated with or triggered by ER stress in rainbow trout RBCs in an antiviral environment by applying a vaccine stimulus. Specifically, we used 2 types of vaccines: a second-generation vaccine presented as “nanopellet” (NP) and formulated with the recombinant fragment 16 of glycoprotein G of VHSV (NP-GVHSV) (Thwaite et al., 2018), and a third-generation DNA vaccine coding for GVHSV (pmTFP1-GVHSV) (Garcia-Valtanen et al., 2014). To achieve this, we adopted an integrative and comparative strategy, combining molecular biology techniques along with high-content screening methods. Thus, we used 2 complementary approaches harnessing synchrotron radiation (SR): SR-based Fourier transform infrared microspectroscopy (SR-µFTIR) and soft X-ray cryotomography (cryo-SXT). The synchrotron light source is more than 100 times brighter than a conventional globular source, and in combination with a microscope allows studies on the dynamics of biochemical changes at the single-cell level with an optimal signal-to-noise ratio and high spatial resolution (Duncan and Williams, 1983, Sasic et al., 2018). SR-based techniques have powered intensive research in biomedical and life sciences, contributing to a deeper understanding of biological systems (Cao et al., 2022, Dumas et al., 2007). SR-µFTIR is an analytical technique applied to the study of the vibrational fingerprint. Cryo-SXT permits three-dimensional (3D) reconstruction at the nanometer scale of intact cryopreserved cell samples without the need for chemical fixation or physical cutting (Garriga et al., 2021, Muller et al., 2012). This technique has become a powerful tool to visualize biological membranes and understand the morphology of membranous organelles and the inside of cells (Sherman et al., 2016). Undoubtedly, the correlation of information from different techniques provides valuable information that could not be collected individually.

Our observations through SR-µFTIR cell analysis suggested that the most significant biochemical changes after treatment with NP-GVHSV and pmTFP1-GVHSV occur at the protein and lipid levels. Cryo-SXT revealed increased ER volume, formation of vesicular structures (VSs), and changes in the mitochondrial profile of vaccine-treated RBCs. While these changes are related to apparently unconnected cellular functions, they are activated at key points of the immune response to respond to the vaccine stimuli. Activation of these pathways was confirmed via the upregulation of gene expression related to UPR^ER^, autophagy, mitochondrial stress, and antigen presentation, along with vesicle trafficking and internalization. These findings highlight RBCs as ubiquitous key immune regulators that can be exploited for novel prophylactic purposes.

## 2 Material and methods

### 2.1 Animals and ethical statements

Specimens of rainbow trout between 5 and 7 cm were obtained from a commercial VHSV-free farm (Mundova, Río Mundo SLU Fish Farm, Albacete, Spain). Once in the animal facilities of Miguel Hernández University, the fish were kept at 14°C in tanks with a recirculating dechlorinated water system and fed daily.

The experimental animal methods and protocols followed in this study were evaluated and approved by the Research Ethics Committee of the Miguel Hernández University and the Animal Welfare Body. All of the experiments described comply with the Guidelines of the European Union Council (2010/63/EU) and Royal Decree RD 53/2013.

### 2.2 RBC isolation and purification

Peripheral blood was extracted directly from the caudal vein using syringes (NIPRO) after sacrifice of rainbow trout with 0.2 g/L tricaine methanesulfonate (Sigma-Aldrich).

To obtain and purify RBCs, 2 consecutive density gradient centrifugations were performed with Ficoll 1007 (Sigma-Aldrich) at 1600 rpm. The resulting RBC pellet was resuspended in RPMI-1640 (Dutch modification) (Gibco, Thermo Fisher Scientific Inc.) supplemented with 10 % gamma-irradiated fetal bovine serum (FBS) (Sigma-Aldrich), 2 mM L-glutamine (Gibco), 50 μg/mL gentamicin (Sigma-Aldrich), 100 μg/mL streptomycin, 100 U/mL penicillin (Sigma-Aldrich), 1 mM sodium pyruvate (Sigma-Aldrich), and 2 μg/mL fungizone (Sigma-Aldrich). The samples were subjected to 2 additional centrifugations at 1600 rpm to remove residual Ficoll and wash the RBCs. Purified RBCs were stored in 25 cm^2^ flasks in RPMI 10 % FBS medium at 14°C for 24 hours before starting experiments.

### 2.3 Vaccines

Two vaccine treatments were used for *in vitro* and *in vivo* assays: the NP-GVHSV and pmTFP1-GVHSV vaccines. The NP-GVHSV is a second-generation vaccine consisting of the selected antigenic protein of interest, the glycoprotein G of VHSV (strain 07–71) (Uniprot KB P27662). NP-iRFP, an inclusion body made of a nonimmunogenic phytochrome-based near-infrared fluorescent protein (iRFP), was used as a control (Filonov et al., 2011). The term nanopellet (NP) refers to viral proteins nanostructured as bacterial inclusion bodies. The production of both nanostructured constructs was carried out from the transformed clones in *E. coli* following previously described methods (Thwaite et al., 2018, Torrealba et al., 2016). The efficacy of NP-based vaccines against bacterial and viral infections has been demonstrated in fish (Puente-Marin et al., 2019b, Rojas-Pena et al., 2022, Thwaite et al., 2018). The third-generation DNA vaccine pmTFP1-GVHSV encodes blue-green fluorescent protein 1 (mTFP1) fused to the C-terminus of the gpG membrane of VHSV (GVHSV) (GenBank accession A10182.1) (Garcia-Valtanen et al., 2014). The plasmid pmTFP1 (Allele Biotechnology, ABP-FP-TCNCS) (Ai et al., 2006) without the antigen of interest was used as the control. The efficacy of this DNA vaccine has been previously described (Chico et al., 2009, Puente-Marin et al., 2018, Puente-Marin et al., 2019a).

### 2.4 Cell treatments

Rainbow trout RBCs previously purified by Ficoll were stimulated with either the NP-GVHSV or pmTFP1-GVHSV vaccines. RBCs were treated with NP-GVHSV or the control NP (NP-iRFP) at a concentration of 50 μg/mL (Puente-Marin et al., 2019b). RBCs were electroporated with pmTFP1-GVHSV or pmTFP1 (control plasmid) using the Neon transfection system (Invitrogen, Thermo Fisher Scientific, Inc.). For each electroporation reaction, 4 μg of plasmid construct (pmTFP1 or pmTFP1-GVHSV) was used per 1 × 10^6^ cells. After suspension in T-buffer (Transfection System Kit) (Invitrogen, Thermo Fisher Scientific, Inc.), samples were electroporated with 1 pulse of 3 ms at 1600 V (Puente-Marin et al., 2018). Vaccine-treated RBCs were seeded in 24-well plates and incubated under the conditions indicated for each experiment.

For cryo-SXT and SR-μFTIR analysis, RBCs treated with pmTFP1-GVHSV and NP-GVHSV at the concentrations indicated above were incubated at 14°C for 1 day and 24 hours, respectively. RBCs were then provided to the ALBA Synchrotron facility.

For temporal screening to identify UPR^ER^ modulators' gene expression, pmTFP1-GVHSV-treated RBCs were incubated for 1, 3, and 6 days and NP-GVHSV-treated RBCs were incubated for 3, 6, 9, and 24 hours. Both vaccine-treated RBCs samples were stored at −80°C in TRK lysis buffer (Omega Bio-Tek, Inc.) for RNA extraction and subsequent quantitative real-time PCR (qPCR) analysis. qPCR analysis was carried out according to the previously described procedure (Nombela et al., 1958) and is detailed below. The primers used to evaluate UPR^ER^ genes can be found in Table 2.

For ER stress inhibition, RBCs were incubated in the presence or absence of 8 mM of 4-phenylbutyric acid (4-PBA) (Sigma-Aldrich) for 24 hours prior to treatment with pmTFP1-GVHSV or NP-GVHSV. RBCs were also incubated in the presence or absence of specific inhibitors for each UPR^ER^ branch: Ceapin-A7 (ATF6 inhibitor, at 12.5 µM final concentration; MedChemExpress); 4µ8C (IRE1α inhibitor, at 10 µM final concentration; MedChemExpress), or ISRIB trans-isomer (PERK inhibitor, at 200 nM final concentration; MedChemExpress). After 24 hours (for Ceapin-A7 and 4µ8C treatments) and 1 hour (for ISRIB treatment), RBCs were subsequently exposed to pmTFP1-GVHSV or NP-GVHSV. Sampling was performed 24 hours after the vaccine treatments and stored in TRK lysis buffer for RNA extraction and qPCR analysis. The primers for UPR^ER^ and autophagy genes analyzed are indicated in Table 2.

### 2.5 Cytotoxicity evaluation of ER stress and UPR^ER^ -related inhibitors

The cytotoxic effect of the ER stress inhibitor (4-PBA) and UPR^ER^ inhibitors (Ceapin-A7, 4µ8C, and ISRIB trans-isomer) on RBCs viability was evaluated by flow cytometry analysis. RBCs were incubated in the presence or absence of 4-PBA (8 mM), Ceapin-A7 (12.5 µM), 4µ8C (10 µM), or ISRIB (200 nM) for the maximum exposure time (inhibitor treatment plus vaccine treatment). As a positive cell death control, untreated RBCs were exposed to 50 % hydrogen peroxide (H_2_O_2_) during 10 min. Next, 1 mg/mL of propidium-iodide (PI) (Sigma-Aldrich) staining was added to the cells. To discriminate between live and dead RBCs, the fluorescence intensity of PI was measured using a FACS Canto II flow cytometer (BD Biosciences).

### 2.6 Cryo-SXT and tomographic reconstruction

Cryo-SXT is the only imaging modality that provides 3D information at the nanometer scale of cryopreserved and unstained whole cells 1–10 μm thick. The penetrating power of soft X-rays (∼0.1–1 keV) includes the spectral region known as the "water window" (between 284 and 543 eV), in which water (dominated by oxygen) is transparent and carbon-rich structures (proteins, lipids, membranes) absorb the X-rays with good contrast. To avoid radiation damage to the samples, hydrated and cryopreserved biological samples can be visualized without the need for additional contrast agents or immobilization in resin. This not only reduces the time required for sample preparation, but also allows the acquired signal to be used quantitatively to determine the densities of the cellular components, and thus qualitatively differentiate them from each other (Carzaniga et al., 2013, Chiappi et al., 2016, Groen et al., 2019, Pereiro, 2019).

To prepare samples, the experimental workflow started with the seeding of vaccine-treated RBCs on quantifoil gold R 2/2 perforated carbon film microscopy grids (Au-G200F1) (Quantifoil Micro Tools GmbH) pretreated with poly-L-lysine (Merck) to facilitate cell adhesion. Cells on the grids were then vitrified by immersion freezing in a Leica EM-CPC using nitrogen-cooled liquid ethane. The quality of freezing and the condition of the cells were examined on a Zeiss Axio Scope fluorescence microscope (Carl Zeiss) using a Linkam cryo-stage. The grids were made up of squares, and each square was identified by an alphanumeric code to identify the regions of interest during data collection. After vitrification, the cryofixed grids were transferred to MISTRAL beamline (ALBA-Light Source, Spain) under cryogenic conditions (Pereiro et al., 2009). During cryo-SXT, the samples were acquired using a Fresnel zone plate objective with an outermost zone width of 40 nm and an effective pixel size of 13 nm at 520 eV photon energy. Weiner deconvolution was applied to the normalized data set to improve image quality. Naper's logarithm was used for linear absorption coefficient (LAC) reconstruction. The output stacks were loaded into (Kremer et al., 1996) IMOD software (RRID: SCR_003297), and alignment and reconstruction of the images for each tilt angle were performed as described previously (Perez-Berna et al., 2016). Finally, visualization, segmentation, and quantification of the final volumes were performed in Amira software (Thermo Fisher, RRID: SCR_007353).

### 2.7 SR-μFTIR measurements and spectra analysis

**Fig. 1:**
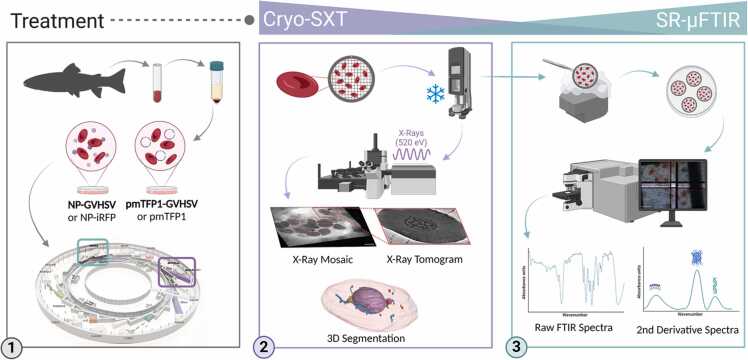
**Systematic workflow of RBC sample preparation and processing for cryo-SXT and SR-µFTIR**. 1) Ficoll-purified RBCs treated with both types of vaccines and their controls were provided to the ALBA Synchrotron facility. 2) Vaccine-treated RBC samples and their respective controls were seeded on quantifoil gold R 2/2 grids and vitrified in a Leica EM-CPC. Then, the samples were loaded on the transmission soft X-ray microscope (MISTRAL beamline). The collected data from cryo-SXT were transformed by reconstruction algorithms into 3D projections that were analyzed with the Amira software. 3) After cryo-SXT analysis, RBCs on R2/2 grids were fixed with PFA and loaded on the µFTIR spectrometer (MIRAS beamline) for single-cell spectra acquisition. For spectra analysis, OPUS and Quasar software were used. The graphic was created with BioRender.

By employing cryogenic conditions, SR-μFTIR enables the analysis of the same samples measured by cryo-SXT without further sample processing. Vaccine-treated RBCs, previously seeded on quantifoil cryofixed grids, underwent a dipping process, beginning with immersion in liquid nitrogen within a 3.7 % paraformaldehyde (PFA) (Sigma-Aldrich) solution at room temperature for 30 minutes. Samples were then dipped 3 times into double-distilled water and dried before measurement (Groen et al., 2021). IR spectra were recorded at the MIRAS beamline (ALBA-Light Source, Spain). Fig. 1 depicts a representative schema of sample preparation for the SR-based methods analysis. Spectral measurements were performed on a Hyperion 3000 IR microscope with a 36 × magnification objective, coupled to a Vertex 70 v FTIR spectrometer (Bruker) and a liquid nitrogen cooled Mercury Cadmium Telluride (MCT) detector. Spectra data were collected in transmission mode at a spectral resolution of 4 cm^−1^ using a 10 μm × 10 μm aperture and a 5 μm step size within the 3700 −1000 cm^−1^ mid-infrared range (Ducic et al., 2022). Each spectrum consisted of 1024 co-added scans. The OPUS 8.2 software package (Bruker, RRID: SCR_025806) was used for data acquisition.

To analyze the data, comparative analyses of pmTFP1-GVHSV vs. pmTFP1 and NP-GVHSV vs. NP-iRFP treatments were conducted using the Quasar software package (Bioinformatics Laboratory of the University of Ljubljana, RRID: SCR_025807) (Toplak et al., 2021). The spectra obtained from the text files were combined and organized into a data table. To compare the pmTFP1 and pmTFP1-GVHSV treatments, 132 and 154 spectral data points were obtained, respectively. Similarly, for the NP-GVHSV vs. NP-iRFP comparison, 183 and 109 spectral data points were obtained, respectively. The data were standardized, and any noisy spectra were removed, resulting in 30 spectra for pmTFP1 and 29 for pmTFP1-GVHSV, and 25 and 19 spectra for NP-GVHSV and NP-iRFP, respectively.

Subsequently, spectral analysis was concentrated on 2 regions based on the specific vibrational modes observed in biological samples (Kreuzer et al., 2020): 1480–1800 cm^−1^ (proteins) and 2800–3000 cm^−1^ (lipids). SR-μFTIR spectra were transformed into a second derivative using the Savitzky-Golay algorithm with 13-point filter and a polynomial order of 3, and vector normalized. The preprocessed data sets were implemented for a multivariate analysis, including PCA, to observe the trend of discrimination between control and vaccine-treated RBCs, and ratio calculation to semiquantitatively assess the relative changes in biomolecular composition and conformation. The relationships between the absorbances of selected peaks used for ratiometric measurements associated with biological processes are shown in Table 1.

**Table 1:** Peak intensity ratios of molecular components derived from SR-µFTIR spectra and their biological assignments (Kreuzer et al., 2020, Nesic et al., 2022, Ricciardi et al., 2020, Saeed et al., 2015). Abbreviations: ν = vibration, as = asymmetric, s = symmetric.

Band assignment	Ratio	Biological indicator
∼1656 / ∼2930	Amide I / ν_as_(CH_2_)	Protein/lipid content; membrane organization
∼2854 / ∼2875	ν_s_(CH_2_) / ν_s_(CH_3_)	Lipid chain packing
∼2930 / ∼2962	ν_as_(CH_2_) / ν_as_(CH_3_)	Fatty acid chain length; lipid saturation level

### 2.8 Immunization assays

Before immunization, fish were anesthetized with 40 mg/L tricaine methanesulfonate. Immunization was carried out by intraperitoneal injection of 100 μg of NP-GVHSV (Puente-Marin et al., 2019b) or NP-iRFP and by intramuscular injection of 10 μg of pmTFP1 or pmTFP1-GVHSV (Puente-Marin et al., 2019a). The indicated amount of each vaccine was resuspended in 50 μL of phosphate buffered saline. At 2 days post-immunization (dpi) for the NP vaccine and 5 dpi for the DNA vaccine, fish were sacrificed by overexposure to 0.2 g/L tricaine methanesulfonate, peripheral blood was drawn, and RBCs were purified as detailed above. Ficoll-purified RBCs were stored in TRK lysis buffer (Omega Bio-Tek Inc.) until RNA extraction for gene expression analysis by qPCR.

### 2.9 Co-culture assays

**Fig. 2:**
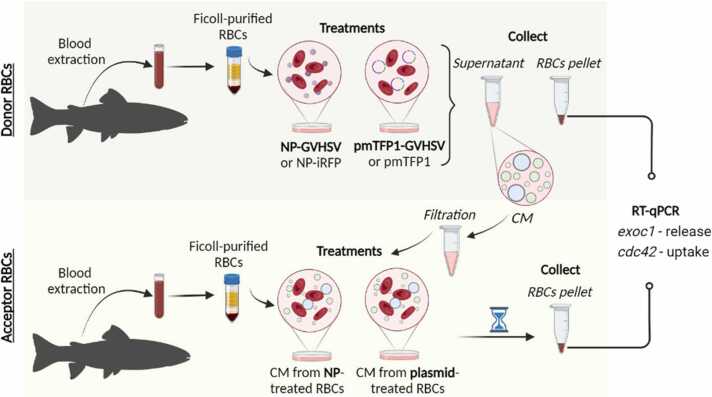
**Schema representing the co-culture assay to evaluate RBCs communication via vesicle trafficking in response to the two types of vaccines.** The treatment for donor RBCs is shown on the top. RBCs were treated with NP-GVHSV, pmTFP1-GVHSV or their respective controls. Supernatants (i.e., conditioned medium [CM]) were filtered. The treatment for acceptor RBCs is shown on the bottom. RBCs were treated with the conditioned medium of donor RBCs. The cell pellets from both treatments were stored until RT-qPCR analysis. The graphic was created with BioRender.

Co-culture assays were performed to evaluate RBC communication via vesicle trafficking in response to vaccine treatment. Communication was evaluated in donor RBCs (vaccine-treated RBCs) and acceptor RBCs (RBCs treated with conditioned medium from donors) (Fig. 2). Ficoll-purified RBCs (5 ×10^5^ cells/well) were treated with NP-GVHSV or NP-iRFP (control), at a concentration of 40 μg/mL and incubated for 48 hours at 14°C. Then, the conditioned medium was collected from each well. Ficoll-purified RBCs (1 ×10^6^ cells/well) were transfected by electroporation with 4 μg of pmTFP1-GVHSV or pmTFP1 (control). The electroporation conditions are described above. Then, RBCs were maintained for 6 days at 14°C. Conditioned medium was collected from the donor RBCs, and filtered through a pore size of 0.2 μm (Cultek) to remove cellular debris and other components secreted into the medium that could interfere with the experiment. Conditioned medium was stored at −80°C until use. RBC pellets were resuspended in TRK lysis buffer and stored at −80°C until RNA extraction. Ficoll-purified RBCs from different individuals were treated with 1/5 and 1/125 dilutions of the conditioned medium of donor RBCs and incubated for 6 hours at 14°C. After this time, acceptor RBCs were stored at −80°C in TRK lysis buffer for RNA extraction and subsequent qPCR analysis.

### 2.10 RNA extraction and cDNA synthesis

RNA was extracted according to the manufacturer's instructions using the E.Z.N.A. Total RNA Kit I (Omega Bio-Tek, Inc.). After RNA extraction, samples were treated with the TURBO DNA-free kit (Invitrogen, Thermo Fisher Scientific Inc.) to remove genomic DNA residue. RNA was quantified with a NanoDrop spectrophotometer (Nanodrop Technologies). M-MLV reverse transcriptase (Invitrogen, Thermo Fisher Scientific Inc.) was used to obtain cDNA as previously described (Chico et al., 2006).

### 2.11 qPCR gene expression analysis

After cDNA synthesis, qPCR was performed using QUANTSTUDIO 3 System (Applied Biosystems, Thermo Fisher Scientific, Inc.). Gene expression was analyzed by the 2^−^^ΔCt^ or 2^−ΔΔCt^ method (Livak and Schmittgen, 2001) as indicated in each experiment, and the eukaryotic translation elongation factor 1 alpha gene (*ef1α*) was used as an endogenous control. Primers and probes are listed in Table 2. The number of samples analyzed in each experiment is indicated by "n" in the figure legends.

**Table 2:** Sequences of primers and probes used in qPCR assays.

Gene	Forward primer (5’-3’)	Reverse primer (5’-3’)	Probe (5’-3’)	Reference or accession number
*atf4*	GGATCAAGAGGTCATGGTGAAGT	GCTCGAGTGTATACCCCATGGA		(Salvador-Mira et al., 2024)
*atf6*	GCCCCAGACCTCGACTTTG	GTCCACACTCAGATCCCCATCT		(Salvador-Mira et al., 2024)
*calr*	GACTGTGGCGGCGGATAT	GCGAGTCTCCGTGCATAGC		(Salvador-Mira et al., 2024)
*cdc42*	GACAGGTTACGGCCTCTGAGTT	AACCCACTTTTCTTTTACGTTTTCG		XM_021609823.2
*cd83*	TTGGCTGATGATTCTTTCGATATC	TGCTGCCAGGAGACACTTGT	TCCTGCCCAATGTAACGGCTGTTG	(Ortega-Villaizan et al., 2012)
*chop*	TTCCTCTCCTGTCTCCTCTCTTACTAG	AGAGTTGCCTCTCTTGCGTTTG		(Salvador-Mira et al., 2024)
*dnm2*	GTCAACAAGTCCATCAGGGATCT	CAACTCAGAATGGATGAAGTCTTTAGC		(Puente-Marin et al., 2018)
*edem1*	CGACCTGTCACCCTGTGAGA	TCCGGTCACAGTTGCTATTGTT		(Salvador-Mira et al., 2024)
*ef1α*	ACCCTCCTCTTGGTCGTTTC	TGATGACACCAACAGCAACA	GCTGTGCGTGACATGAGGCA	(Raida and Buchmann, 2007)
*exoc1*	AGCTTATCAGAGCCGTCTTTATGAA	TGGAGAAGATGTGGTGGAAGTTC		(Salvador-Mira et al., 2024)
*gabarap*	CCTCATCCATCCATTTTTACCTCTT	ATTCAACCGAAATCCCC ATCT	TCTGAATTTTATTTGCCTCCGGGTCTCC	(Nombela et al., 2019)
*grp78*	CCCCAGATCGAGGTCACCTT	CTTGTTGCCTGTTCCCTTGTC		(Salvador-Mira et al., 2024)
*mhcII*	TGCCATGCTGATGTGCAG	GTCCCTCAGCCAGGTCACT	CGCCTATGACTTCTACCCCAAACAAAT	(Jorgensen et al., 2009)
*sec13*	GCAGTGATCCAGGCACAGAA	CTGGGACTAGGATAGATGGTAGAAGTG	ATTCCACTCCTCCTCCTACCCCCACA	(Puente-Marin et al., 2018)
*trap1*	ATGGTCCAGAAGTGGCATGTG	GCTTCCTTGGTGCTGTAGTAGGA		(Salvador-Mira et al., 2024)
*ulk1*	CTTCTGCTGCTGGGTCTTCTG	GGTGACGGAAGAACTCCTCAAA	CGAAACCACAAGGACCGCATGGA	(Nombela et al., 2019)
*wipi1*	CAAAGACATGAAGCTG CTGAAGA	GGTTCACAGAGAGGGCACAGA	CTCAACACGCCCCACAACCCCT	(Puente-Marin et al., 2019a)

### 2.12 Analysis of *xbp1* mRNA splicing by semi-quantitative PCR

The cDNA of RBCs from individuals immunized with pmTFP1-GVHSV or NP-GVHSV was analyzed by semi-quantitative PCR (semi-qPCR) to evaluate the splicing of the X-box binding protein 1 gene (*xbp1*) mRNA (XM_036980188.1). *xbp1* primers were designed: 5′-TTCTCTGGTGATGGAACTGGAGA-3′ (forward) and 5′-GCTCTCTGGGTGAAGGATGTCA-3′ (reverse). PCR amplification reactions were performed using the following components: 0.5 μL of 10 mM dNTP mix (Invitrogen, Thermo Fisher Scientific), 0.125 μL GoTaq DNA polymerase (Promega Biotech), 5 μL 5X Green GoTaq reaction buffer (Promega Biotech), 0.5 μL of each primer (20 μM), and 2.5 μL cDNA, in a final reaction volume of 25 μL. Amplification was performed on a GeneAmp PCR System 2700 thermocycler (Applied Biosystems, Thermo Fisher Scientific). The cycling conditions were: 1 cycle of 94°C for 5 minutes, followed by 40 amplification cycles at 95°C for 30 seconds, 52°C for 30 seconds, and 72°C for 30 seconds. Finally, 1 extension cycle was performed at 72°C for 7 minutes. PCR products were resolved on a 2 % agarose gel (Pronadisa, Laboratorios Conda S.A.), stained with GelRed nucleic acid dye (Biotium, Inc.), and visualized with the Chemidoc XRS transilluminator (Biorad). For semi-qPCR analysis of *xbp1* expression, we assessed the band intensity of unspliced *xbp1* (*xbp1*^*U*^) and spliced *xbp1* (*xbp1*^*S*^) and calculated the ratio *xbp1*^*S*^/ *xbp1*^*U*^ to estimate cellular *xbp1* splicing activity*.*

### 2.13 Software and statistical analysis

GraphPad Prism 8 (Graphpad Software Inc., RRID: SCR_002798) was used for statistical analysis and the resulting graphical representation. The *P* values associated with each assay are shown in figure captions. ClustVis software (https://biit.cs.ut.ee/clustvis/) was used for PCA and gene expression clustering (Metsalu and Vilo, 2015). For cytotoxicity assays, Flowing Software 2.5.1 (www.flowingsoftware.com/, RRID: SCR_015781) was employed to analyze flow cytometry data, and Floreada.io software (https://floreada.io/, RRID: SCR_025286) was used to generate histograms and dot plots. Scion Image software (Scion Corp., RRID: SCR_008673) was used for band densitometry and quantification.

## 3 Results

### 3.1 Vaccine treatments induced biochemical changes in rainbow trout RBCs

Vibrational spectra are a fundamental and highly informative tool for the study of biochemical changes. RBCs are ideal subjects for SR-μFTIR because the microscope aperture is smaller than the cell diameter, effectively minimizing Mie scattering (Webster et al., 2009). This study was the first to use SR-μFTIR to evaluate the impact of vaccine stimulation on the biochemical properties of teleost RBCs. We obtained absorption spectra of single intact cryopreserved RBCs treated with either a second-generation (NP-GVHSV) or third-generation (pmTFP1-GVHSV) vaccine. RBCs treated with NP-iRFP or pmTFP1 were used as respective controls. First, the average raw (unprocessed) spectra of stimulated RBCs were plotted to analyze the biochemical composition and distribution of major macromolecules. The resulting SR-µFTIR spectra were divided into 2 main zones (between 3000 and 1450 cm^−1^) with different visible bands due to protein and lipid contributions (Supplementary Figure S1). Vaccine-treated RBCs had distinct spectral features compared to control RBCs. The protein amide I band at ∼1656 cm^−1^ and amide II band at ∼1545 cm^−1^ were the most pronounced bands in the spectra, while symmetric and asymmetric CH_2_ and CH_3_ stretching vibrations dominated the CH stretching region between 2800 and 3000 cm^−1^. These differences were more clearly visible after computing second derivative spectra.

**Fig. 3:**
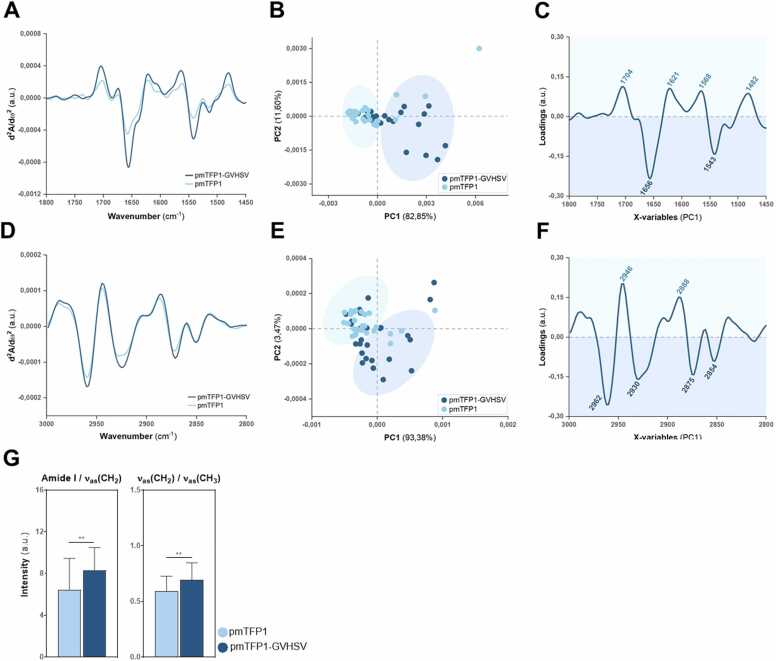
**SR-µFTIR analysis of biochemical characteristics of RBCs treated with pmTFP1-GVHSV.** Averaged second derivative spectra of the (A) protein and (D) lipid regions of RBCs when treated with pmTFP1-GVHSV or pmTFP1 (control). PCA score plots for (B) protein and (E) lipid regions and corresponding PCA loading plots (PC1) for (C) protein and (F) lipid regions. (G) Mean peak intensity ratios of amide I/ν_as_(CH_2_) (protein-to-lipid ratio) and ν_as_(CH_2_)/ν_as_(CH_3_) (lipid ratio) from the second derivative spectra. Box plots represent mean ± standard deviation. The non-parametric Mann-Whitney test was used for comparison between treatments (**P < 0.01). The total number of spectra analyzed was 29 for pmTFP1-GVHSV-treated RBCs and 30 for control RBCs. Frequency ranges are measured as wavenumbers typically over the range of 1800–1450 cm^−1^ for proteins and 3000–2800 cm^−1^ for lipids. Dark blue and light blue correspond to pmTFP1-GVHSV-treated RBCs and control RBCs, respectively.

**Fig. 4:**
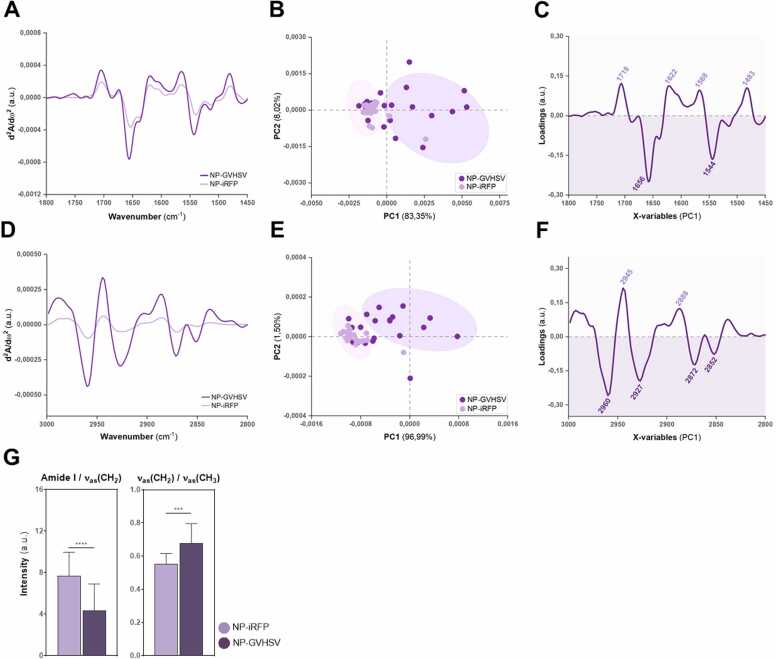
**SR-µFTIR analysis of biochemical characteristics of RBCs treated with NP-GVHSV.** Averaged second derivative spectra of the (A) protein and (D) lipid regions of RBCs when treated with NP-GVHSV or NP-iRFP (control). PCA score plots for (B) protein and (E) lipid regions and corresponding PCA loading plots (PC1) for (C) protein and (F) lipid regions. (G) Mean peak intensity ratios of amide I/ν_as_(CH_2_) (protein-to-lipid ratio) and ν_as_(CH_2_)/ν_as_(CH_3_) (lipid ratio) from the second derivative spectra. Box plots represent mean ± standard deviation. The non-parametric Mann-Whitney test was used for comparison between treatments (***P < 0.001; ****P < 0.0001). The total number of spectra analyzed was 25 for NP-GVHSV-treated RBCs and 19 for control RBCs. Frequency ranges are measured as wavenumbers typically over the range of 1800–1450 cm^−1^ for proteins and 3000–2800 cm^−1^ for lipids. Dark purple and light purple correspond to NP-GVHSV-treated RBCs and control RBCs, respectively.

The second derivative spectra of vaccine-treated RBCs showed some shifts for protein and lipid regions regarding wavenumber and differences in the maximum absorbance compared to control RBCs. Within the protein region (1800–1450 cm^−1^), differences were detected in the curve fit for amide I and amide II band contours in pmTFP1-GVHSV-treated RBCs (Fig. 3A) and NP-GVHSV-treated RBCs (Fig. 4A) compared to their respective controls. Substantial variations in the intensities of amide I and II bands (∼1656 cm^−1^ and ∼1545 cm^−1^) were recorded for both treatments. Within the lipid region spectra (3000–2800 cm^−1^), changes in peak intensities were observed in RBCs stimulated with pmTFP1-GVHSV or NP-GVHSV compared to control RBCs (Fig. 3D and Fig. 4D, respectively). Additional modifications in pmTFP1-GVHSV-treated RBCs were observed in absorption peaks at ∼2925 and ∼2960 cm^−1^, corresponding to the CH_2_ and CH_3_ asymmetric vibrational groups ([ν_as_(CH_2_)] and [ν_as_(CH_3_)], respectively) (Fig. 3D). Symmetric and asymmetric vibrations of CH_2_ and CH_3_ displayed a sharp increase in peak intensities in NP-GVHSV-treated RBCs compared to the control (Fig. 4D).

Next, a multivariate analysis was conducted to determine the spectral covariance between the different treatment groups for the 2 spectral regions of interest. Principal component analysis (PCA) was carried out on normalized second derivative spectra (Rieppo et al., 2012). Score plots were constructed for the first 2 principal components (PC1 and PC2) to assess sample clustering. The loadings were used in combination with the scores plot to identify the spectral variables (wavenumber values) into which the different sample groups can be distinguished. The scores and loadings in PCA are related, so positive scores are associated with negative loadings and vice versa because of the second derivative (Perera et al., 2021).

Both vaccine-treated RBCs and their respective controls were well discriminated in the score plots of the protein region along PC1, accounting for more than 80 % of the total components (Fig. 3B and Fig. 4B). Notably, RBCs treated with pmTFP1-GVHSV or NP-GVHSV were grouped at the positive extremes on the PC1 axis of the score plots, while the controls were grouped at the negative extremes. The corresponding PC1 loading plots were used to identify key differences in the biochemical composition that are responsible for the separation. Negative loadings in the protein region loading plots represented key differences for vaccine-treated RBCs, and positive loadings referred to those for control RBCs (Fig. 3C and Fig. 4C). Strong negative loadings at the amide I (∼1656 cm^−1^) and amide II (∼1543 cm^−1^) bands contributed to the separation of vaccine-treated RBCs (pmTFP1-GVHSV or NP-GVHSV) from their controls along the PC1 scores plot. The identified peaks had little significance in the separation of groups for the positive loadings (Fig. 3C and Fig. 4C). Thus, variance along PC1 was due to changes in protein concentration or composition of mainly the amide I and amide II bands in vaccine-treated RBCs.

In score plots for the lipid region, pmTFP1-GVHSV-treated RBCs were largely distinguished from the control by PC1—although not symmetrical around the origin—with some discriminability by PC2 (Fig. 3E). Similarly, RBCs treated with NP-GVHSV were discriminated from control RBCs by PC1, which contributed to ∼97 % of the observed variance (Fig. 4E). According to the most prominent component loadings for the lipid region along PC1, vaccine-treated RBCs displayed strong negative loadings centered at ∼ 2962 cm^−1^ and ∼ 2930 cm^−1^, assigned to the asymmetric stretching of CH_3_ and CH_2_, and also at ∼2875 cm^−1^ and ∼2854 cm^−1^, corresponding to CH_3_ and CH_2_ symmetric stretching (Fig. 3F and Fig. 4F). Positive loadings showed two bands centered at ∼2946 cm^−1^ and ∼2888 cm^−1^ (Fig. 3F and Fig. 4F). These findings suggested that vaccine-treated RBCs differed from controls regarding the amount of lipids.

Because vibrational frequencies differ depending on the concentration of the molecular structures associated with the band, we took relative measurements between the maximum absorption of selected peaks. This ratiometric approach allows assessment of the most influential biochemical changes in vaccine-treated RBCs. These ratios and their biological assignments are shown in Table 1. Values obtained for RBCs treated with pmTFP1-GVHSV or NP-GVHSV and their controls were plotted in Fig. 3G and Fig. 4G, respectively. The protein-to-lipid ratio (PLR), here amide I/ν_as_(CH_2_), describes the change in lipid vs. protein content and may be a useful indicator of biological membrane function. RBCs treated with pmTFP1-GVHSV had an increased PLR compared to control RBCs (Fig. 3G). Conversely, a decreased PLR was detected for NP-GVHSV-treated RBCs relative to their control (Fig. 4G). Thus, the protein concentration was higher in pmTFP1-GVHSV-treated RBCs while NP-GVHSV-treated RBCs had a higher lipid concentration, implying changes in membrane composition between the vaccine types.

The effect of vaccine treatments on cellular lipids was further evaluated via lipid ratio (LR), here ν_as_(CH_2_)/ν_as_(CH_3_). CH_2_ vibrations are mainly related to saturated lipid chains, and CH_3_ vibrations are mostly associated with the contribution of methyl groups in lipids and proteins. Changes in these ratios imply alterations in the degree of saturation and lipid chain length (Nesic et al., 2022, Saeed et al., 2015). Upon treatment with pmTFP1-GVHSV or NP-GVHSV, the value of ν_as_(CH_2_)/ν_as_(CH_3_) significantly increased, revealing that fatty acid chains became more saturated than in control RBCs (Fig. 3G and Fig. 4G). These data reflect an effect on the RBC lipid profile of as a result of vaccine treatments, which correlates with the PLR calculated for NP-GVHSV but not for pmTFP1-GVHSV.

### 3.2 Morphometric assessment revealed ER expansion and mitochondrial and vesicular formation in vaccine-treated RBCs

We used cryo-SXT, a near-native 3D imaging technique, to directly visualize the subcellular reorganization and regulatory effects induced in RBCs by vaccine treatments. The 3D reconstructed tomographies revealed that pmTFP1-GVHSV and NP-GVHSV treatments induced appreciable intracellular changes in rainbow trout RBCs relative to the control treatments (pmTFP1 and NP-iRFP).

**Fig. 5:**
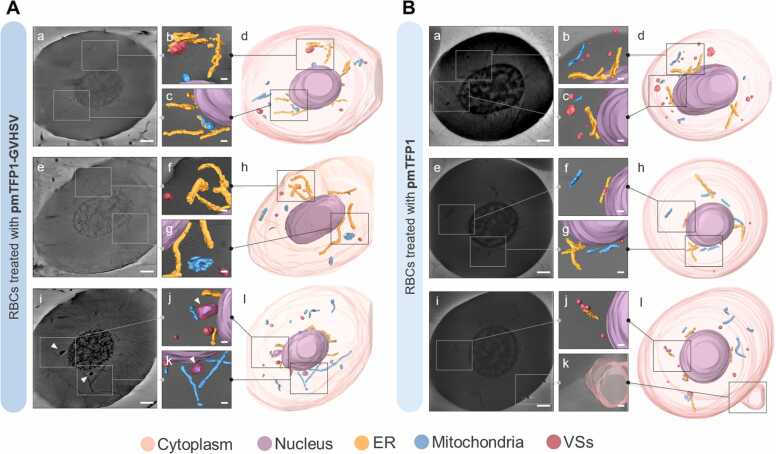
**3D ultrastructural analysis of rainbow trout RBCs in response to pmTFP1-GVHSV treatment via cryo-SXT.** Tomograms and 3D reconstructions of (A) pmTFP1-GVHSV-treated RBCs and (B) pmTFP1-treated RBCs (control). For each reconstruction, 2D orthoslices (a, e, and i) and 3D-rendered images (d, h, and l) of the segmentations showing the whole RBC, as well as scale-up 2D/3D overlays of the desired sections (b-c, f-g, and j-k) are shown. White arrows indicate vesicles containing plasmid DNA. The images were reconstructed and rendered using Amira software to show cellular structures: cytoplasm (translucent pink); nucleus (purple); ER (yellow); mitochondria (blue); and VS (red). Scale bars 100 nm (a, e, and i); and 50 nm (b-c, f-g, and j-k).

**Fig. 6:**
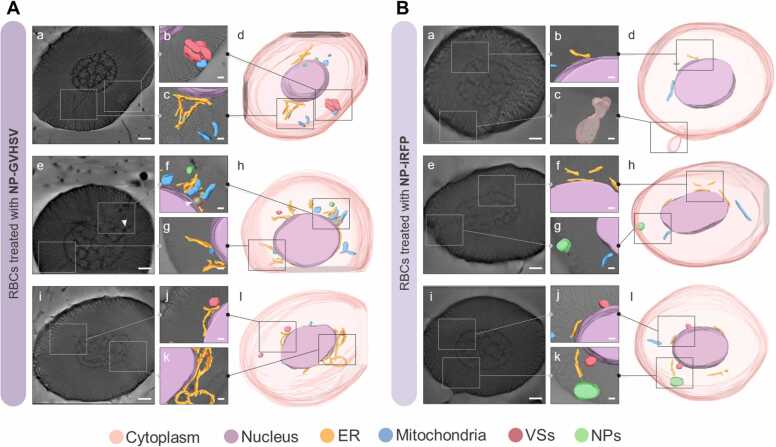
**3D ultrastructural analysis of rainbow trout RBCs in response to NP-GVHSV treatment via cryo-SXT.** Tomograms and 3D reconstructions of (A) NP-GVHSV-treated RBCs and (B) NP-iRFP-treated RBCs (control). For each reconstruction, 2D orthoslices (a, e, and i) and 3D-rendered images of the segmentations showing the whole RBC (d, h, and l), as well as scale-up 2D/3D overlays of the desired sections (b-c, f-g, and j-k) are shown. White arrows indicate vesicles containing NPs. The images were reconstructed and rendered using Amira software to show cellular structures: cytoplasm (translucent pink); nucleus (purple); ER (yellow); mitochondria (blue); VS (red); and NPs (green). Scale bars 100 nm (a, e, and i); and 50 nm (b-c, f-g, and j-k).

**Fig. 7:**
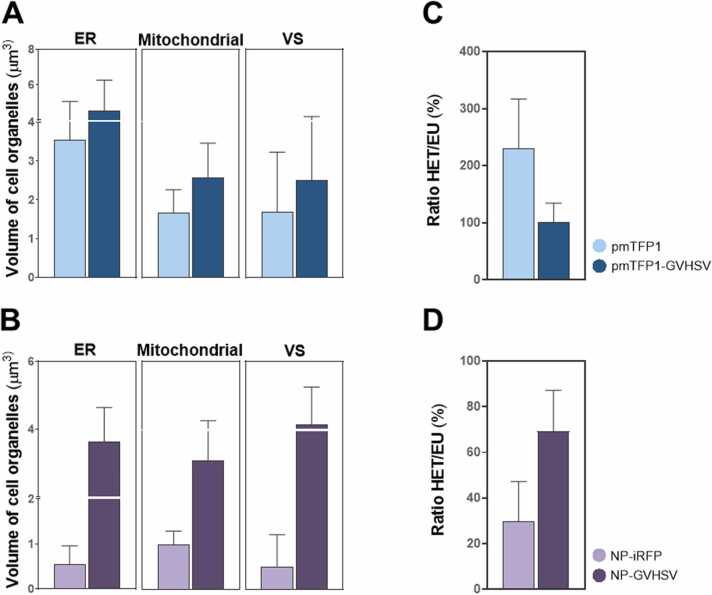
**Volumetric quantification of structural changes in rainbow trout RBCs in response to pmTFP1-GVHSV or NP-GVHSV treatment.** ER, mitochondrial, and VS volume computation plots for (A) pmTFP1-GVHSV-treated RBCs compared to pmTFP1-treated RBCs (control) and (B) NP-GVHSV-treated RBCs compared to NP-iRFP-treated RBCs (control). Graphical representation of the heterochromatin/euchromatin (HET/EU) ratio in (C) pmTFP1-GVHSV-treated RBCs compared to their control and in (D) NP-GVHSV-treated cells compared to their control. Data represent mean ± standard deviation (n = 3). The non-parametric Mann-Whitney test was used for statistical analysis between treatments. All calculations were performed with Thermo Scientific Amira software.

Vaccine stimulation was sufficient to trigger a response capable of functionally activating the ER. ER size was increased in RBCs treated with pmTFP1-GVHSV or NP-GVHSV and, in some cases, markedly thickened (Fig. 5A and Fig. 6A). Volumetric data supported these observations, with a 2-fold increase in pmTFP1-GVHSV-treated RBCs (Fig. 7A) and an almost 4-fold increase in NP-GVHSV-treated RBCs (Fig. 7B) compared to their controls.

Cryo-SXT demonstrated increased mitochondrial number and size in pmTFP1-GVHSV- and NP-GVHSV-treated RBCs compared to their controls (Fig. 5 A and Fig. 6A). Mitochondria typically appear spherocylindrical when located close to the nucleus and undergo a morphological transition to an elongated state as distance increases from the nucleus (Picard, 2015, Prowse et al., 2012). Analysis showed a 3-fold increase in the mitochondrial volume of pmTFP1-GVHSV-treated samples (Fig. 7A) and a 2-fold increase in NP-GVHSV-treated samples (Fig. 7B) compared to their controls. Further, it could be appreciated that mitochondria established contact with the ER in RBCs treated with both types of vaccines (Fig. 5A-c,d,f and Fig. 6A-c,d). In contrast, the mitochondria in control RBCs were located close to the nucleus, and had no apparent contact with other organelles. No clear pattern of morphology and shape was observed in pmTFP1-GVHSV- and NP-GVHSV-treated RBCs. In pmTFP1-GVHSV- and NP-GVHSV-treated RBCs, we found mitochondria with an elongated appearance, even forming clusters or extensive reticular structures (Fig. 5A-g,h,k,l and Fig. 6A-f,h). Notably, we frequently observed mitochondria and VSs surrounding the ER because of the expanded cisternal design of the ER in vaccine-treated RBCs (Fig. 5A-d and Fig. 6A-d).

We observed a higher number of and larger VSs in pmTFP1-GVHSV- and NP-GVHSV-treated RBCs compared to their controls (Fig. 5A and Fig. 6A). VSs were located closer to the ER and mitochondria in pmTFP1-GVHSV- or NP-GVHSV-treated RBCs (Fig. 5A-b,d and Fig. 6 A-b,d,k,l), which could indicate that VSs derive from these organelles. Further, NP-GVHSV-treated RBCs showed an association between a multivesicular body and a mitochondrion (Fig. 6A-b). Volumetric analysis of the VSs showed a 2-fold increase in volume in pmTFP1-GVHSV-treated RBCs (Fig. 7A) and a 4-fold increase for NP-GVHSV-treated RBCs (Fig. 7B). Additionally, we observed the formation of vesicles containing DNA-like material in pmTFP1-GVHSV-treated RBCs, though this material may have derived from pmTFP1-GVHSV due to the high absorbance of DNA at 520 eV (Fig. 5A-i,j,k). Vesicles containing NP particles were also identified in NP-GVHSV-treated RBCs presumably due to their high protein content and round shape (Fig. 6A-e,f).

We calculated the heterochromatin/euchromatin (HET/EU) ratio by analyzing nucleus fractions and compared the volume, absorbance, and shape between the different treatments. Each voxel in the cryo-SXT reconstructed volume corresponds to the LAC or absorbance of the material within it (Carzaniga et al., 2013, Groen et al., 2019). The absorbance, which translates to the density of the analyzed nuclear fractions, can then be compared. A bimodal distribution can be readily observed inside the nucleus as clear zones (lower absorbance) and darker regions (higher absorbance) (Groen et al., 2021). Analysis of LAC values revealed the heterochromatin/euchromatin distribution. Heterochromatin, which is a tightly packed condensed form of DNA with less transcriptional activity, presented the highest LAC values. While euchromatin, which is a lightly packed form of the DNA in an open state and therefore more accessible to the transcriptional machinery (Morrison and Thakur, 2021), had lower LAC values, suggesting lower condensation of chromatin (Smith et al., 2014). Low HET/EU ratios correspond to more transcriptionally active cells, and high ratios correspond to lower cellular transcriptional activity. Our results showed that NP-GVHSV-treated RBCs had a higher HET/EU ratio than control RBCs (Fig. 7D), indicating lower transcriptional activity in NP-GVHSV-treated RBCs. In contrast, pmTFP1-GVHSV-treated RBCs had a lower HET/EU ratio compared to their control (Fig. 7C), indicating increased transcriptional activity in pmTFP1-GVHSV-treated cells.

### 3.3 Dynamic modulation of the UPR^ER^ in vaccine-treated RBCs

We investigated the underlying mechanism behind ER expansion after vaccine treatment by analyzing the expression patterns of major transducers and mediators of the UPR^ER^ pathways at different time points in RBCs *ex vivo*. These UPR^ER^ transducers and mediators include: (1) *grp78*, an essential ER chaperone and master regulator of the UPR^ER^; (2) the gene encoding transcription factor C/EBP homologous protein (*chop*), which is a UPR^ER^ mediator of the PERK/eIF2α branch; (3) *calreticulin* (*calr*), a quality control chaperone regulated by the ATF6 branch of the UPR^ER^; and (4) the ER degradation enhancing alpha-mannosidase-like protein 1 (*edem1*) gene, a key component in the ER quality-control system that functions as a downstream effector target of the IRE1α branch.

**Fig. 8:**
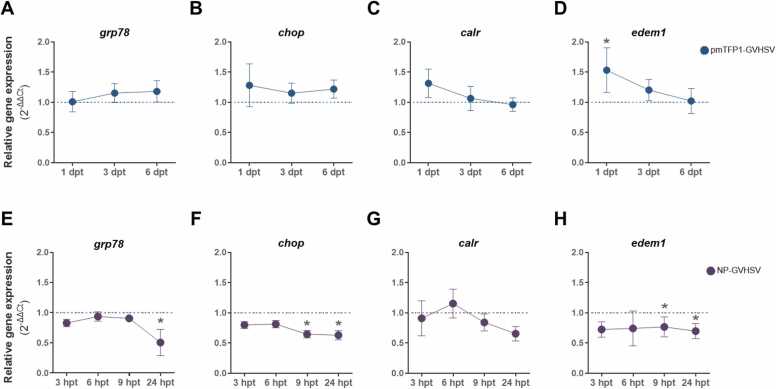
**Temporal screening to identify UPR**^**ER**^**modulators from candidate genes in rainbow trout RBCs in response to vaccine treatment.** Expression of (A) *grp78*, (B) *chop*, (C) *calr*, and (D) *edem1* in RBCs transfected with pmTFP1-GVHSV at 1, 3, and 6 days post-treatment (dpt). Expression of (E) *grp78*, (F) *chop*, (G) *calr*, and (H) *edem1* in NP-GVHSV-treated RBCs at 3, 6, 9, and 24 hours post-treatment (hpt). *ef1α* was used as the endogenous gene. Gene expression is presented as relative expression levels (2^-ΔΔCt^) compared to control RBCs. Plotted data represent the mean ± standard deviation (n = 3). Statistical analysis was performed using the Kruskal-Wallis test with Dunn's multiple comparison test. *P < 0.05 relative to the control.

Expression profiles of the major UPR^ER^ genes and their downstream targets vary between both types of vaccine stimulation. In RBCs treated with pmTFP1-GVHSV, transcriptional modulation of *grp78* and *chop* was mild and non-significantly increased at the time points analyzed (Fig. 8A,B). However, *calr* and *edem1*, the downstream targets of the UPR^ER^ transducers ATF6 and IREIα, respectively, appeared overexpressed at 1 day post-treatment (dpt), being statistically significant in the case of *edem1* (Fig. 8C,D), suggesting an implication of the ATF6 and IREIα/XBP1-mediated UPR^ER^ pathways in RBCs after pmTFP1-GVHSV stimulation.

In contrast, in RBCs treated with NP-GVHSV, the expression levels of *grp78* and the genes involved in the PERK/eIF2α and IREIα/XBP1 UPR^ER^ branches, *chop* and *edem1* (Fig. 8E,F,H), were significantly downregulated in a time-dependent manner compared to control RBCs. Nevertheless, *calr* expression was slightly induced at 6 hours post-treatment (hpt) with NP-GVHSV, although not statistically significant (Fig. 8G).

The differences observed in the modulation of the UPR^ER^ indicated that the vaccine stimulus and the timing of activation play crucial roles in the direction of this RBCs response.

### 3.4 Inhibition of ER stress differentially regulated UPR^ER^ and autophagy

To evaluate the effect of ER stress inhibition on UPR^ER^ in vaccine-treated RBCs, cells were pretreated with 4-PBA at a final concentration of 8 mM for 24 hours before vaccine treatment. The ER stress inhibitor 4-PBA is a chemical chaperone that helps to stabilize unfolded proteins and facilitates their proper folding (Yam et al., 2007). Prior to the experiment, we determined that 4-PBA did not have a cytotoxic effect on RBCs at the selected dose (Supplementary Figure S2).

**Fig. 9:**
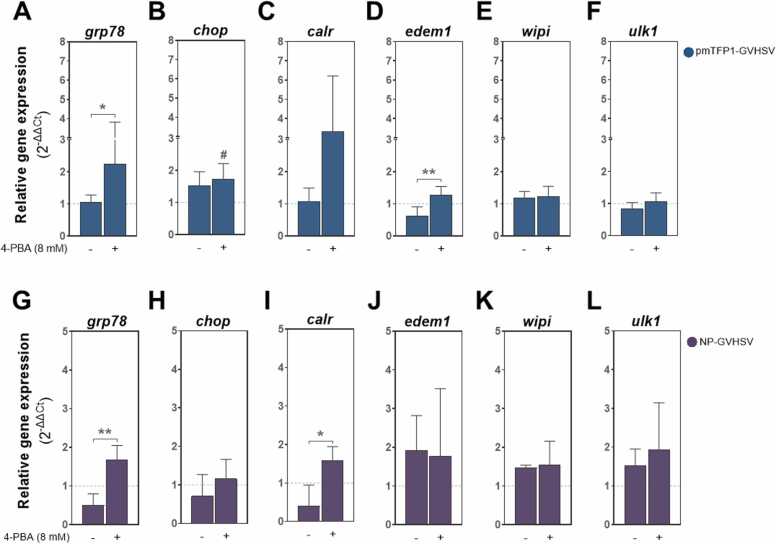
**Effect of ER stress inhibition by 4-PBA on UPR**^**ER**^**and autophagy-related genes in rainbow trout RBCs in response to vaccine treatment.** RBCs were exposed to 8 mM 4-PBA for 24 hours, followed by treatment with pmTFP1-GVHSV or NP-GVSHV. Expression levels of *grp78*, *chop*, *calr*, *edem1, wipi1*, and *ulk1* in pmTFP1-GVHSV-treated RBCs (A, B, C, D, E, and F, respectively) and NP-GVHSV-treated RBCs (G, H, I, J, K, and L, respectively). *ef1α* was used as the endogenous gene. Gene expression is presented as relative expression levels (2^-ΔΔCt^) compared to control RBCs (pmTFP1- and NP-iRFP-treated RBCs with or without 4-PBA). Plotted data represent the mean ± standard deviation (n = 3). Statistical analysis was performed using the Kruskal-Wallis test with Dunn's multiple comparison test. #*P* < 0.05 relative to the control. *P < 0.05 and * *P < 0.01 between treatments.

In the presence of 4-PBA, *grp78* expression significantly increased in vaccine-treated RBCs compared to vaccine-treated RBCs in the absence of 4-PBA (Fig. 9A,G). Moreover, the effect of 4-PBA resulted in an upregulation of *calr* in vaccine-treated RBCs, being statistically significant in the case of NP-GVHSV treatment (Fig. 9C,I). Notably, *edem1* was upregulated only in pmTFP1-GVHSV-treated RBCs (Fig. 9D). Because 4-PBA reduces ER stress and UPR^ER^ activation without directly inhibiting the specific sensors of this response, we further tested specific inhibition of the UPR^ER^ branches using Ceapin-A7, 4µ8 C or ISRIB, which selectively inhibit the ATF6, IRE1α or PERK pathways, respectively (Cross et al., 2012, Gallagher et al., 2016, Zyryanova et al., 2021) (Supplementary Figure S3). Notably, none of the inhibitors compromised RBC viability (Supplementary Figure S2). In pmTFP1-GVHSV-treated RBCs, none of these inhibitors significantly altered the expression of the target genes of each UPR^ER^ pathway (Supplementary Figure S3 B). On the other hand, while the expression of UPR^ER^-related genes was not affected by 4µ8 C or ISRIB inhibitors in NP-GVHSV-treated RBCs, selective inhibition of ATF6 with Ceapin-A7 resulted in a marked upregulation of *grp78*, *atf6*, and *calr* (Supplementary Figure S3 B), aligning with the upregulation observed with 4-PBA treatment. These results indicated a differential regulation of ER stress responses between both types of vaccines.

By modulating ER stress, 4-PBA can influence autophagic pathways, either promoting or inhibiting autophagy depending on the cellular context (Wang et al., 2020, Zeng et al., 2017). To understand how inhibiting ER stress impacts autophagy in vaccine-treated RBCs, we assessed the gene expression of important autophagy effectors, such as WD repeat domain phosphoinositide-interacting protein 1 (*wipi1*) and uncoordinated 51-like kinase 1 (*ulk1*). Interestingly, ER stress inhibition by 4-PBA did not alter the regulation of *wipi1* and *ulk1* in vaccine-treated RBCs (Fig. 9E,F,K,L), and only *ulk1* expression was slightly, non-significantly increased in the case of NP-GVHSV-treated RBCs (Fig. 9L).

Collectively, these results imply a dual role of 4-PBA for UPR^ER^ and autophagy in vaccine-treated RBCs. While the inhibition of ER stress by 4-PBA paradoxically led to increased activation of UPR^ER^ signaling pathways when combined with vaccine stimulus, autophagy remained unchanged.

### 3.5 Vaccine immunization promotes the activation of UPR^ER^, autophagy, and related cellular mechanisms

**Fig. 10:**
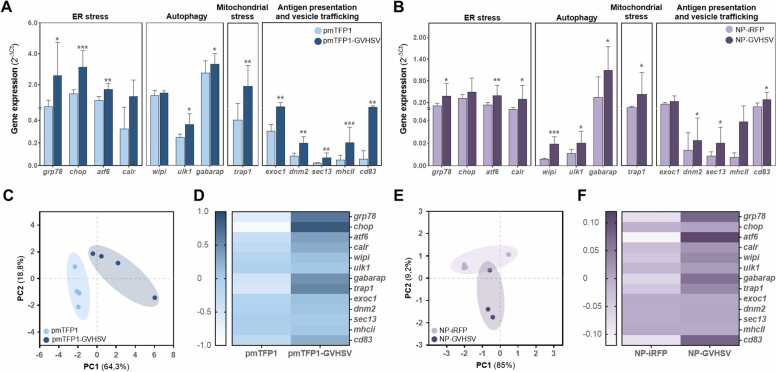
**Cellular processes in RBCs from rainbow trout immunized with pmTFP1-GVHSV or NP-GVHSV**. Expression analysis of genes related to ER stress, autophagy, mitochondrial stress, antigen presentation, and vesicle trafficking evaluated in RBCs from rainbow trout injected with (A) pmTFP1-GVHSV or pmTFP1 (control), at 5 dpi and (B) NP-GVHSV or NP-iRFP (control), at 2 dpi. Data represent absolute gene expression (2^-ΔCt^) normalized using *ef1 α* as the endogenous gene. Bars indicate mean ± standard deviation (n = 4). The non-parametric Mann-Whitney test was used for statistical analysis between treatments (*P < 0.05; **P < 0.01; ***P < 0.001). Multivariate analysis of the gene expression, including (C, E) PCA plots of molecular markers (gene expression results, 2^-ΔCt^) and (D, F) heatmaps of the molecular markers (gene expression results, 2^-ΔCt^). Population clustering in the PCA plots is represented by ellipses, and dots indicate biological replicate values.

We hypothesized that UPR^ER^ triggered by ER stress may act in concert with signaling pathways involved in other cellular processes to orchestrate the RBC immune response. This is supported by the findings in vaccine-treated RBCs of substantial changes in ER shape, the range of mitochondrial morphological subtypes observed, increased vesicle formation, and close contact between these organelles. To this end, we investigated how immunization with second- or third-generation vaccines modulates cellular pathways critical for cellular homeostasis and immune response in RBCs by quantifying gene transcript levels (Fig. 10). Selected genes were organized into clusters: UPR^ER^ (*grp78, chop*, *atf6* and *calr*); autophagy (*wipi1*, *ulk1*, and gamma-aminobutyric acid receptor-associated protein [*gabarap*]); mitochondrial stress (tumor necrosis factor receptor-associated protein 1 [*trap1*]); antigen presentation and vesicle trafficking (exocyst complex component 1 [*exoc1*]; dynamin 2 [*dnm2*]; SEC13 homolog, nuclear pore and COPII coat complex component [*sec13*]; major histocompatibility complex class II [mhcII]; and cluster of differentiation 83 [cd83] (De Leo et al., 2021, Gonzalez-Jamett et al., 2013, Hara et al., 2008, Hetz, 2012, Kuwano et al., 2007, Niu et al., 2012, Slobodkin and Elazar, 2013, Takamura et al., 2012, Wiederkehr et al., 2003).

We investigated the transience and rapidity of how immunization induces ER stress and regulates UPR^ER^ signaling pathways. Transcriptional expression of *grp78* was upregulated in RBCs from rainbow trout immunized with pmTFP1-GVHSV or NP-GVHSV. Notably, *grp78* expression showed a statistically significant increase of almost 4-fold with pmTFP1-GVSHV immunization and 3-fold with NP-GVHSV immunization (Fig. 10A,B). Analogously, we detected statistically significant overexpression of *atf6* by up to 3-fold and 2-fold in RBCs from rainbow trout immunized with pmTFP1-GVHSV or NP-GVHSV, respectively, along with 3-fold (nonsignificant) and 5-fold increase in *calr* transcript levels. In contrast, *chop* expression was upregulated by 2-fold increase with pmTFP1-GVHSV immunization but showed no change with NP-GVHSV immunization. In addition, we evaluated the implication of IREIα/XBP1 pathway in RBCs after immunization, via *xbp1* splicing. Upon activation, IRE1α cleaves a 26-nucleotide (nt) intron in *xbp1* mRNA, resulting in a frameshift (Calfon et al., 2002, Yoshida et al., 2001). Conversion of *xbp1*^*U*^ to *xbp1*^*S*^ form at the mRNA level was evaluated on agarose gels by semi-qPCR (Supplementary Figure S4 A,B). Results did not show *xbp1* splicing in RBCs from immunized fish (pmTFP1-GVHSV or NP-GVHSV), as the densitometric analysis of the *xbp1*^*S*^/*xbp1*^*U*^ ratio remained unchanged compared to their controls (Supplementary Figure S4 C,D).

Since ER stress and autophagy are closely related, we explored the modulation of *wipi1*, *ulk1*, and *gabarap*, because of their key roles at different stages of the autophagy process. All three genes were significantly upregulated in RBCs under NP-GVHSV immunization (Fig. 10B). *ulk1* and *gabarap*, but not *wipi1*, were significantly increased by 2-fold in RBCs following pmTFP1-GVHSV immunization (Fig. 10A).

Mitochondria share signaling mechanisms with the ER, and like the ER, mitochondria have stress response mechanisms to regulate protein quality control (Hori et al., 2002, Takemoto et al., 2011). These findings, plus the ER-mitochondria linkage that was seemingly established in the tomographies of vaccine-treated RBCs, compelled us to evaluate *trap1* gene expression. The expression of *trap1* demonstrated a statistically significant increase in RBCs from individuals immunized with pmTFP1-GVHSV or NP-GVHSV, by up to 3-fold and 2-fold, respectively, compared to their controls (pmTFP1 or NP-iRFP) (Fig. 10A,B).

We also investigated whether signals from intracellular components beyond the mitochondria and ER contribute to induction of the UPR^ER^. For example, vesicles store and transport molecules with known biological functions to enhance the response, and antigen presentation plays a role in this task. For this purpose, we evaluated expression of the following genes: *sec13*, a constituent of the ER that mediates vesicle biogenesis and vesicle trafficking to the Golgi apparatus (Kaiser and Ferro-Novick, 1998); *dnm2,* a GTPase involved in the final step of endocytosis and in the creation of new vesicles from the Golgi apparatus (Gonzalez-Jamett et al., 2013); *exoc1*, a component of the exocyst complex involved in vesicle secretion and release (Wiederkehr et al., 2003); and *mhcII* and *cd83*, genes related to antigen presentation (Klein et al., 2005, Kuwano et al., 2007, Roche and Furuta, 2015). Statistically significant upregulation of expression was detected for *exoc1*, *dnm2*, *sec13*, *mhcII*, and *cd83* with pmTFP1-GVHSV immunization and *dnm2*, *sec13*, and *cd83* with NP-GVHSV immunization (Fig. 10A,B).

PCA analysis showed clearly differentiated populations for each treatment (Fig. 10C,E), and the heatmap displayed a sharp enrichment mainly consisting of *grp78*, *cd83*, *gabarap1*, *trap1*, and *atf6* overexpression in RBCs from individuals immunized with pmTFP1-GVHSV or NP-GVHSV (Fig. 10D,F).

### 3.6 Vesicle trafficking participates in cellular communication between RBCs in response to vaccine treatment

**Fig. 11:**
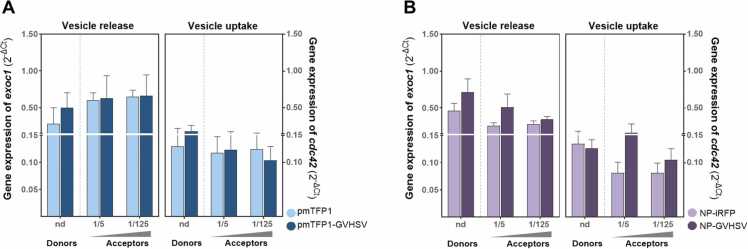
**Vesicle trafficking-related gene expression involved in RBC communication triggered by vaccine treatment**. Vesicle release and vesicle uptake during (A) pmTFP1-GVHSV or (B) NP-GVHSV treatments between donor and acceptor RBCs. Donor RBCs were treated with pmTFP1-GVHSV or pmTFP1 (control) for 6 days or with NP-GVHSV or NP-iRFP (control) for 48 hours. Acceptor RBCs were treated with conditioned medium from donor RBCs at 2 dilutions (1/5 and 1/125) for 6 hours. ‘nd’ indicates no dilution. Data represent absolute gene expression (2^-ΔCt^) and normalized using *ef1 α* as the endogenous gene. Data mean ± standard deviation (n = 3). A Kruskal-Wallis test with Dunn's multiple comparison test was used for statistical analysis between treatments.

Research in mammals has established that some vesicles released into the extracellular matrix originate from RBCs and may even interact with nearby cells (Arraud et al., 2014). However, the role of vesicles and their trafficking in fish RBCs is not known. Because RBCs stimulated with either the pmTFP1-GVHSV or NP-GVHSV vaccines demonstrated an increased number of VSs in the tomographies and because contact with other organelles was also observed, we examined the regulation of genes involved in vesicle trafficking mechanisms (Fig. 11). To this end, we analyzed gene expression of *exoc1*, a mediator of vesicular secretion and exocytosis, and cell division cycle 42 (*cdc42*), a manager of endocytic activity and vesicle uptake, between donor and acceptor RBCs. "Donor" refers to RBCs that were treated with pmTFP1-GVHSV or NP-GVHSV and their respective control treatments (pmTFP1 and NP-iRFP). "Acceptor" refers to RBCs that received the conditioned medium from donor RBCs.

We detected upregulation of *exoc1* and *cdc42* in donor RBCs treated with pmTFP1-GVHSV compared to the control (pmTFP1 treatment) (Fig. 11A). Only *exoc1* expression was increased in NP-GVHSV-treated RBCs compared to the control (NP-iRFP treatment) (Fig. 11B). Among acceptor RBCs, while *exoc1* and *cdc42* expression was unchanged in acceptor RBCs treated with conditioned medium derived from pmTFP1-GVHSV treatment at both dilutions, stimulation with NP-GVHSV-derived conditioned medium resulted in upregulation of *exoc1* and *cdc42* expression at a 1/5 dilution (Fig. 11A,B).

## 4 Discussion

Autophagy and ER stress help keep the cell in proper internal equilibrium, by removing misfolded proteins and damaged or unneeded organelles. However, many viruses exploit both processes to survive and replicate in host cells. Noteworthy, Nombela and colleagues (Nombela et al., 2019) demonstrated the implication of autophagy and antigen degradation pathways in the antiviral immune response of RBCs. Although little is known about this role for ER stress, we recently identified that VHSV leads to ER enlargement in RBCs, a hallmark of heightened ER stress and subsequent UPR^ER^ activation. Notably, VHSV replication levels augmented in RBCs under ER stress inhibition, suggesting that rainbow trout RBCs tune up ER stress to control viral replication (Salvador-Mira et al., 2024). The present study provides a new concept of ER stress as a mandatory cellular response of RBCs in the context of vaccination. Our findings establish that vaccine treatment modulates ER stress by UPR^ER^ regulation. This response is integrated into cooperating cellular networks (such as autophagy, mitochondrial dynamism, and vesicle trafficking) and have been proposed to act as signaling hubs for regulating the RBC antiviral immune response. The submicron resolution provided by cryo-SXT and SR-µFTIR allowed us to explore biochemical and structural processes in a single RBC after stimulation with a DNA vaccine (pmTFP1-GVHSV) or subunit vaccine based on a nanostructured recombinant antigen (NP-GVHSV). To our knowledge, the present work is the first study of the intracellular behavior of fish RBCs using correlative cryo-SXT and SR-µFTIR techniques relying on synchrotron radiation.

Our results showed that RBCs treated with GVHSV antigen-based vaccines underwent changes in protein content and lipid content and composition, including variations in saturation degree and acyl chain length, as shown by SR-µFITR. We discovered a significant contribution of lipid CH stretching bands in vaccine-treated RBCs. The most substantial variations were found in the CH_2_ and CH_3_ asymmetric bands. This, together with a significant increase in the ν_as_(CH_2_)/ν_as_(CH_3_) ratio, suggested changes in RBC lipid content and saturation with both vaccine treatments. Further, the PLR suggested increased lipid content for NP-GVHSV-treated RBCs and a higher protein content for pmTFP1-GVHSV-treated RBCs (possibly attributed to plasmid-encoded GVHSV protein synthesis). Lipids have two vital responsibilities in cells. First and foremost, they are the main building blocks of cells, providing the basic structure of the cell membrane and subcellular compartments (e.g., ER, Golgi, vesicles and mitochondria). Secondly, they serve as energy reserves (Cooper, 2000, Holthuis and Menon, 2014). Proteins are contained within membranes and constitute approximately half the mass of most cell membranes. We hypothesize that the changes in lipid and protein content that we observed could be explained by the accumulation and expansion of suborganelles within the cell, since cell membrane integrity must remain stable to maintain RBC structure and function. Overall, RBCs membrane dynamics may be adaptively remodeled based on the needs of the cell (Szalontai et al., 2000, Tsvetkova et al., 2002). Further, lipids act as messenger molecules that transmit signals from cell surface receptors to intracellular targets (Prokazova et al., 2007), and recent studies have detailed how lipid secondary messengers can stimulate the innate immune response (Flores et al., 2000, Poerio et al., 2017). These examples contextualize a broader discussion of the role of lipids and proteins as central regulators of innate immune signaling pathways in RBCs.

We found that RBCs experienced ER thickening after vaccine treatment, as demonstrated by cryo-SXT. Interestingly, other studies reported altered 3D ER architecture in a human hepatoma cell line during hepatitis C virus infection (Groen et al., 2021, Perez-Berna et al., 2016). Further, rainbow trout IgM^+^ B cells experience ER expansion—relevant to their differentiation process—upon stimulation with a pathogen-associated molecular pattern such as lipopolysaccharide (Morel et al., 2023). The ER is the principal factory of lipid and protein synthesis in the cell. Larger ER volume is indicative of higher membrane lipid and protein production. The activation of lipid biosynthesis drives ER membrane expansion (Holthuis and Menon, 2014, Schuck et al., 2009), and changes in membrane dynamics or lipid saturation in turn activate ER stress sensors (Garcia et al., 2023, Volmer et al., 2013). As such, membrane expansion is governed by ER sheet formation, UPR^ER^ signaling, and lipid biosynthesis. Nevertheless, we cannot determine whether the lipid changes we detected by SR-µFTIR are caused by or an effect of ER membrane expansion and UPR^ER^ induction upon vaccine treatment in RBCs. However, SR-µFITR and cryo-SXT findings in vaccine-stimulated RBCs suggest interdependence between protein and lipid content within ER enlargement. In this context, the UPR^ER^ is crucial to alleviate ER stress.

Some studies support the concept that UPR^ER^ signaling follows a temporal pattern with specific outcomes depending on the cellular needs, the stimulus, and the duration of stimulation (DuRose et al., 2006, Rutkowski et al., 2006). In rainbow trout RBCs, *grp78* and *chop* remained relatively stable under both vaccine treatments while *calr*, a chaperone that assists in proper protein folding within the ER, appeared slightly modulated in response to both types of vaccines. Enhanced levels of *edem1* in pmTFP1-GVHSV-treated RBCs, but not in NP-GVHSV-treated RBCs, could be associated with accelerated degradation of misfolded glycoproteins (Olivari et al., 2006). CALR is activated under ER stress by the accumulation of misfolded proteins in the ER (Yang et al., 2019) and is a downstream effector of the UPR^ER^ mediated by the ATF6 pathway (Schardt et al., 2008). EDEM1 is a target gene of the IREIα/XBP1 pathway but also can be activated by ATF6 (Yamamoto et al., 2007). Of note, the transcription factor ATF6 participates in the activation of genes that expand ER size in order to increase protein folding and enhance cell survival (Maiuolo et al., 2011, Oakes and Papa, 2015). As such, ATF6 regulation seems sufficient to induce a protective ER stress response that drives ER expansion in vaccine-treated RBCs. We hypothesize that as cells constantly adjust their organelle size and shape on demand, the UPR^ER^ adjusts ER stress through 2 distinct but interdependent mechanisms: by providing new ER folding machinery and more ER surface area to host ER chaperones (Schuck et al., 2009). Even without robust induction of UPR^ER^ -related genes at the time points analyzed, a more subtle and balanced response may reflect a lower level of ER stress or faster resolution of misfolded proteins.

Although *in vitro* assays revealed only partial activation of the UPR^ER^ transcription program with a focus on the ATF6 branch, *in vivo* experiments provided more compelling evidence of UPR^ER^ engagement by overexpression of the stress sensor *grp78* and the subsequent signaling network of *atf6*, *calr*, and *chop* in RBCs from vaccine-immunized individuals. Thus, the simultaneous upregulation of *atf6* and *calr* supports the notion that the ATF6 branch of the UPR^ER^ is engaged to enhance ER capacity for protein folding and processing in RBCs. CHOP is a multifunctional transcription factor that plays a variety of roles in determining cell fate depending on the intensity and duration of ER stress (Southwood et al., 2016). CHOP has been widely associated with the induction of apoptosis, but in certain contexts, such as with anticipatory UPR^ER^ or specific stressors, it may not lead to apoptosis and can instead be involved in other cellular responses such as autophagy (B'Chir et al., 2013). Thus, CHOP may function as part of a broader adaptive response of the UPR^ER^ in RBCs, triggered by vaccination.

When we attempted to suppress ER stress by 4-PBA, UPR^ER^-related genes such as *grp78* and *calr* were unexpectedly upregulated in vaccine-treated RBCs. Generally, 4-PBA affects the cell in several ways, including histone deacetylase inhibition and prevention of accumulated misfolded proteins in the ER by UPR^ER^ attenuation (Lea et al., 1999, Yam et al., 2007). However, the target and mechanism of action of 4-PBA are still uncertain. It is worth noting that we are not the first to fail in detecting chaperone activity with 4-PBA treatment (Mai et al., 2018, Stein et al., 2022). We speculate that 4-PBA was not able to restore global protein folding status in RBCs, possibly because the vaccine stimulus resulted in an overload in the ER lumen that the UPR^ER^ must resolve. This protein turnover, together with heightened UPR^ER^ activation, may at least partially counteract the effect of 4-PBA in suppressing ER stress. While 4-PBA was used as a broad ER stress inhibitor, we next sought to dissect the specific contribution of each UPR^ER^ branch by using selective inhibitors targeting ATF6 (Ceapin-A7), IRE1α (4μ8 C), or PERK (ISRIB trans-isomer) (Han et al., 2024, Hu et al., 2023, Sidrauski et al., 2015, Zhang et al., 2014). Rather than suppressing the pathway, ATF6 inhibition led to a paradoxical overexpression of *atf6* and its downstream targets in NP-GVHSV-treated RBCs, a similar effect as the observed with 4-PBA treatment, suggesting a compensatory mechanism to counteract the burden of misfolded proteins. This overexpression appeared to mitigate the inhibitory effect of Ceapin-A7, reinforcing the hypothesis that the ATF6-mediated UPR^ER^ pathway is an important regulatory step in managing ER stress in RBCs in response to NP vaccine. In contrast, inhibition of IRE1α and PERK had no significant impact in NP-GVHSV-treated RBCs, and none of these inhibitors affected UPR^ER^ modulation in pmTFP1-GVHSV-treated RBCs. These findings highlight a possible antigen-dependent nature of UPR^ER^ activation in RBCs. Further studies are needed to explore whether these differences translate into functional consequences for RBCs and their immune-related roles.

It is worth noting that tunicamycin, a well-known ER stress inducer, did not affect ER stress-related genes at low concentrations in either NP-GVHSV- or pmTFP1-GVHSV-treated RBCs, while higher concentrations were cytotoxic (data not shown), precluding further analysis.

Other signaling pathways might play a role in managing ER stress to deal with the accumulated unfolded proteins. Autophagy can serve as an adaptive stress response that helps to sustain cell homeostasis, just as the UPR^ER^ may be effective in manipulating autophagy (Rashid et al., 2015). However, when we attempted to suppress ER stress by 4-PBA, genes associated with autophagy, such as *wipi1* and *ulk1*, were not affected. The lack of effect on autophagy genes indicates that autophagy might be regulated independently of the UPR^ER^ in this specific context and could require another form of stress or signaling for activation. Further protein-focused experiments may help to clarify this.

On the other hand, vaccine immunization with pmTFP1-GVHSV or NP-GVHSV modulated key autophagy effectors in RBCs. The pmTFP1-GVHSV vaccine resulted in the upregulation of *ulk1* and *gabarap*, whereas NP-GVHSV vaccine elicited a broader autophagic response, characterized by upregulation of *ulk1*, *gabarap*, and *wipi1*, possibly due to a greater accumulation of misfolded proteins. Proteins encoded by these genes play distinct yet collaborative roles at different stages of the autophagy process: ULK1 initiates the process (Hara et al., 2008), WIPI1 helps to scaffold and regulate autophagosome formation (De Leo et al., 2021), and GABARAP facilitates cargo transfer and ULK1 activation (Slobodkin and Elazar, 2013). Overall, induction of these autophagy effectors suggests that autophagy, similar to the UPR^ER^, contributes to the RBCs response to both types of vaccines.

Cryo-SXT tomographies of vaccine-treated RBCs showed mitochondrial enlargement and morphological diversity, changing from rounded to long, tube-like structures that weave through the cytosol. Glancy and colleagues described how mitochondrial phenotype can change in response to stimuli (Glancy et al., 2020). Several mechanisms, including mitochondrial biogenesis, fusion, and fission, are crucial for regulating mitochondrial dynamics, heterogeneity, and ultimately cellular homeostasis. Other cellular components, such as the ER, influence mitochondrial architecture through interorganelle contact sites that modulate mitochondrial biochemical composition, function, and dynamics (Abrisch et al., 2020). Furthermore, the the ER-associated protein degradation (ERAD) pathway regulates mitochondrial size through interorganelle mechanisms (Zhou et al., 2020), which implies a relationship between ER stress and mitochondrial elongation (Zhou et al., 2020). Our cryo-SXT findings revealed what appeared to be a physical linkage, (i.e., contact sites) between the ER-mitochondria surface in RBCs stimulated with pmTFP1-GVHSV or NP-GVHSV, with the physical linkage being more predominant in the latter. Given that some reports suggest that ER stress increases ER-mitochondria relocation and the contact sites between them (Boland et al., 2013, Bravo et al., 2011), we propose that the GVHSV antigen induces ER-mitochondrial transport and/or communication in RBCs. Interestingly, the membrane contact sites (MCSs) that connect the organelles are particularly enriched in specific lipid species (Flis and Daum, 2013). Mitochondrial function depends on a coordinated supply of proteins and lipids, and although they are capable of synthesizing several lipids autonomously, mitochondria depend on the import of certain proteins and lipids from the ER (Daum and Vance, 1997, Rowland and Voeltz, 2012). Precisely, these MCSs between donor (ER) and acceptor (mitochondria)—known as mitochondria-ER contact sites (MERCS)—appear to contribute to lipid transport. While the mechanisms underlying this process are still a matter of debate, this lipid exchange provides a platform for interorganelle communication (Kannan et al., 2017, van Vliet et al., 2014). We examined *trap1* gene expression, which was significantly induced in RBC machinery upon immunization of rainbow trout with pmTFP1-GVHSV or NP-GVHSV. TRAP1 has recently been associated with the UPR^ER^ in the ER under stress conditions and stimulates mitochondrial division (Takamura et al., 2012, Takemoto et al., 2011). TRAP1 has been implicated in the regulation of mitochondrial physiology (Takamura et al., 2012), which is governed by the fine-tuning of mitochondrial fusion/fission (Chan, 2006, Otera and Mihara, 2011). This process is likely responsible for the morphological diversity observed in cryo-SXT images in our study. Gomes and colleagues (Gomes et al., 2011) found that mitochondria acquire this elongated morphology in response to autophagy and to avoid degradation. We have previously shown that autophagy processes were induced in RBCs of rainbow trout in response to VHSV (Nombela et al., 2019) or different types of vaccines (Puente-Marin et al., 2018). Besides, MERCS have been proposed as a mechanism to modulate autophagy and antigen presentation via the MHC pathway (Martinvalet, 2018). This is in line with our findings of overexpression of antigen presentation-related genes and autophagy intermediates in RBCs from immunized rainbow trout. Little is known about the involvement of TRAP1 in ER-mitochondria communication, but *trap1* expression has been linked to the reduction of prolonged ER stress (Kokott-Vuong et al., 2021). Thus, mitochondria could be potential regulators of the UPR^ER^ via TRAP1 in vaccine-treated RBCs. In summary, the ignition and development of the innate and adaptive immune responses of RBCs to the GVHSV antigen require mitochondrial remodeling, autophagy, and antigen processing and presentation, which are dependent on lipid transfer and MERCS.

The exchange of material between organelles is mediated in part by vesicles, whose transport depends on protein-lipid interactions (Muller et al., 2019). Cryo-SXT results showed that stimulation with vaccines led to a vast proliferation of VSs in RBCs, many of which are in close proximity to ER and mitochondria. The ER produces phospholipids to supply the cell's endomembrane system, and once synthesized in the ER, phospholipids are transported to their destinations via vesicles, a process facilitated by MCSs (Jain and Holthuis, 2017, Simmen and Tagaya, 2017). A high percentage of saturated lipids has been detected in secreted vesicles from ZIKV-infected human brain microvascular endothelial cells (Fikatas et al., 2021), similar to what we observed in SR-µFITR spectra from vaccine-treated RBCs, though we cannot confirm the responsible organelle. Higher saturation of lipids is advantageous because it results in increased membrane rigidity and reduces susceptibility to oxidative stress. In turn, this allows vesicles to better transfer and release their contents (Rysman et al., 2010). The ability of VSs to mediate RBC cellular communication has been described in mammals (Buttari et al., 2015), and vesicle trafficking processes and their participation in cellular communication are likely conserved in fish (Nikapitiya et al., 2022). Given the ability of VSs to modulate cell-cell crosstalk, we examined whether conditioned media derived from vaccine-treated RBCs can stimulate vesicle trafficking-related pathways. *exoc1* and *cdc42* were used to track external or internal events in RBCs triggered by components (possibly VSs) contained within conditioned media. *exoc1*, a gene related to vesicle secretion, was mildly upregulated in rainbow trout RBCs following pmTFP1-GVHSV or NP-GVHSV stimulation, though only NP-GVHSV impacted vesicle uptake regulation via *cdc42*, a finding supported by higher vesicle count in cryo-SXT. VSs are versatile effectors, and their action depends on the level of stimulation and the environmental context in which the cell, in this case the RBC, is located. However, experimental findings are limited by the need to purify VSs from vaccine-treated RBCs and assess their cargo.

In our study, we determined the expression level of genes involved in vesicle trafficking, formation, secretion, and uptake (*exoc1*, *dnm2*, and *sec13*) in RBCs from vaccine-immunized fish. We observed significant upregulation of these genes, indicating that the GVHSV antigen stimulates vesicle trafficking mechanisms in RBCs. The regulation of vesicle formation, exocytosis, and endocytosis is mediated by EXOC1 and DNM2. EXOC1 is an exocytic reference point that directs secretory vesicles to specific plasma membrane docking sites for fusion (Wiederkehr et al., 2003), and DNM2 is a central player in endocytosis, responsible for vesicle formation and transport from endosomes and the Golgi apparatus (Gonzalez-Jamett et al., 2013). *dnm2* was significantly upregulated in RBCs from rainbow trout immunized with pmTFP1-GVHSV or NP-GVHSV. However, *exoc1* was mainly upregulated in RBCs from pmTFP1-GVHSV-immunized fish and only slightly upregulated in RBCs from NP-GVHSV-immunized fish. This suggests that both studied vaccines induced the secretion of extracellular vesicles, allowing communication between RBCs. The coat protein complex II (COPII), in which *sec13* participates, is responsible for the transport of newly synthesized proteins from the ER to the Golgi apparatus and their subsequent secretion (Niu et al., 2012). Our results showed increased gene expression of *sec13* in RBCs from pmTFP1-GVHSV- or NP-GVHSV-immunized fish. Raposo and colleagues (Raposo et al., 1996) first reported that mammalian B cells release extracellular vesicles carrying MHCII molecules that are then presented to T cells. This finding, as well as results from other studies, demonstrated the antigen-presenting capacity of VSs with a clear immune component (Qazi et al., 2009, Tung et al., 2018). We investigated the expression of *cd83* and *mhcII*. Both genes were upregulated in RBCs from individuals immunized with pmTFP1-GVHSV, while only *cd83* expression increased in RBCs from individuals immunized with NP-GVHSV. CD83 is a plasma membrane-localized glycoprotein whose expression is positively correlated with MHCII (Kuwano et al., 2007). These findings support previous results in which a DNA or NP vaccine based on GVHSV induced the expression of genes involved in antigen processing and presentation in rainbow trout RBCs (Nombela et al., 2019, Puente-Marin et al., 2019a). Recently, ER expansion was linked to high MHCII levels and antigen processing capacities, which are valuable features for fish IgM^+^ B cells (Morel et al., 2023).

**Fig. 12:**
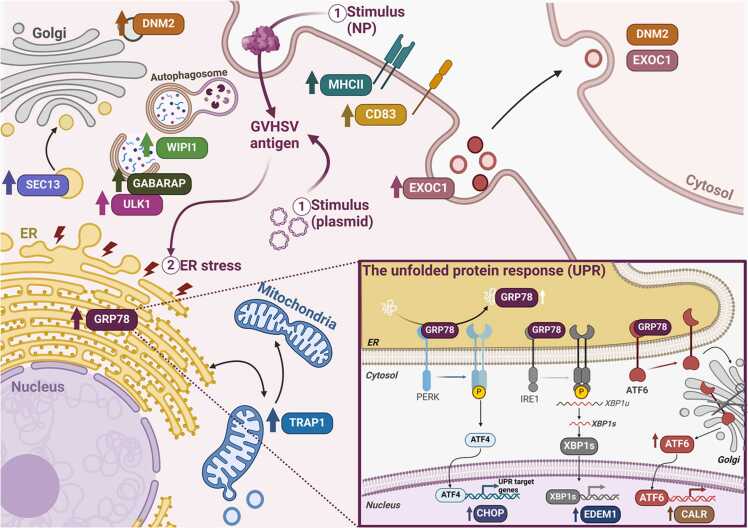
**Proposed scheme of signaling pathways of fish RBCs in response to GVHSV antigen**. Upon vaccine stimulation (step 1), the viral antigen is processed in the cell, causing ER stress (step 2) and activating the UPR^ER^. The ER stress sensor GRP78 dissociates from PERK, IRE1, and ATF6, activating downstream UPR signaling pathways. Concurrently, other signaling pathways are activated to manage the stress response and support the immune response: ULK1, WIPI1, and GABARAP, as autophagy effectors; TRAP1, related to UPR^ER^ and mitochondrial function; SEC13, responsible for protein transport from the ER to the Golgi; MHCII and CD83, involved in antigen presentation and processing; and DNM2 and EXOC1, mediating vesicle trafficking.

Although the detailed mechanism of the UPR^ER^ triggered by ER stress, and its interplay with autophagy, mitochondrial stress, and vesicle trafficking on RBCs immune response, remain unclear, the present findings (Fig. 12) highlight these novel processes as ideal cellular targets for the development of more effective prophylactic tools with greater immunogenic capacity than currently available options.

## CRediT authorship contribution statement

**Salvador-Mira Maria:** Writing – review & editing, Writing – original draft, Validation, Methodology, Investigation, Formal analysis, Conceptualization. **Gimenez-Moya Paula:** Methodology, Investigation, Formal analysis. **Manso-Aznar Alba:** Methodology, Investigation, Formal analysis. **Sánchez-Córdoba Ester:** Methodology, Investigation, Formal analysis. **Sevilla-Diez Manuel A.:** Methodology, Investigation, Formal analysis. **Chico Veronica:** Writing – review & editing, Methodology, Investigation, Formal analysis. **Nombela Ivan:** Methodology, Investigation, Formal analysis. **Puente-Marin Sara:** Methodology, Investigation, Formal analysis. **Roher Nerea:** Writing – review & editing, Resources. **Perez Luis:** Writing – review & editing, Funding acquisition. **Dučić Tanja:** Methodology. **Benseny-Cases Núria:** Writing – review & editing, Formal analysis. **Perez-Berna Ana Joaquina:** Writing – review & editing, Methodology, Formal analysis. **Ortega-Villaizan Maria del Mar:** Writing – review & editing, Supervision, Resources, Methodology, Investigation, Funding acquisition, Formal analysis, Conceptualization.

## Funding statement

This work was supported by grants from the 10.13039/501100000780European Commission (ERC Starting Grant GA639249) to MOV and the Spanish Ministry of Science (RTI2018-096957-B-C22 MINECO/FEDER) to MOV and LP.

## Declaration of Competing Interest

The authors declare that they have no known competing financial interests or personal relationships that could have appeared to influence the work reported in this paper.

## Data Availability

Data will be made available on request.
