# Supplementary material for: Viral vaccines promote endoplasmic reticulum stress-induced unfolding protein response in teleost erythrocytes
Source: Eur J Cell Biol. 2025 Jun;104(2):151490. doi: 10.1016/j.ejcb.2025.151490 (PMC12162347; doi:10.1016/j.ejcb.2025.151490)
Supplement: Supplementary Figures S1, S2, S3, and S4 — Supplementary material [file mmc1.docx]

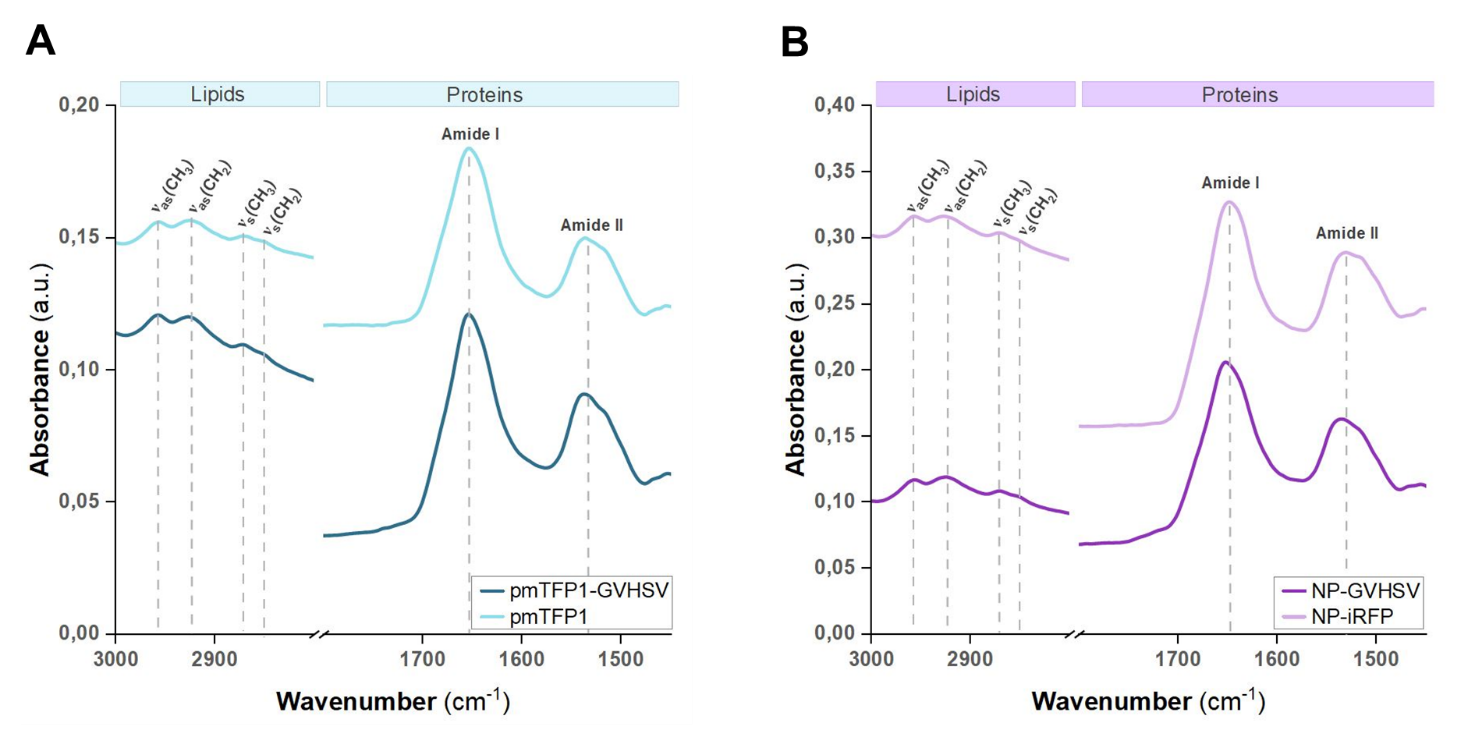
**Supplementary information**

**Figure S1. Averaged absorption spectra of vaccine-treated RBCs (related to Fig. 3 and Fig. 4).** (A) Spectra of RBCs treated with pmTFP1-GVHSV and pmTFP1 (control). (B) Spectra of RBCs treated with NP-GVHSV and NP-iRFP (control). Two regions of interest were identified for further analysis: the spectral range of lipids (3000-2800 cm^-1^) with the absorption bands corresponding to the symmetric and asymmetric stretch of CH_2_ y CH_3_; and the spectral range of proteins (1800-1450 cm^-1^) with the absorption bands corresponding to amide I and amide II groups.


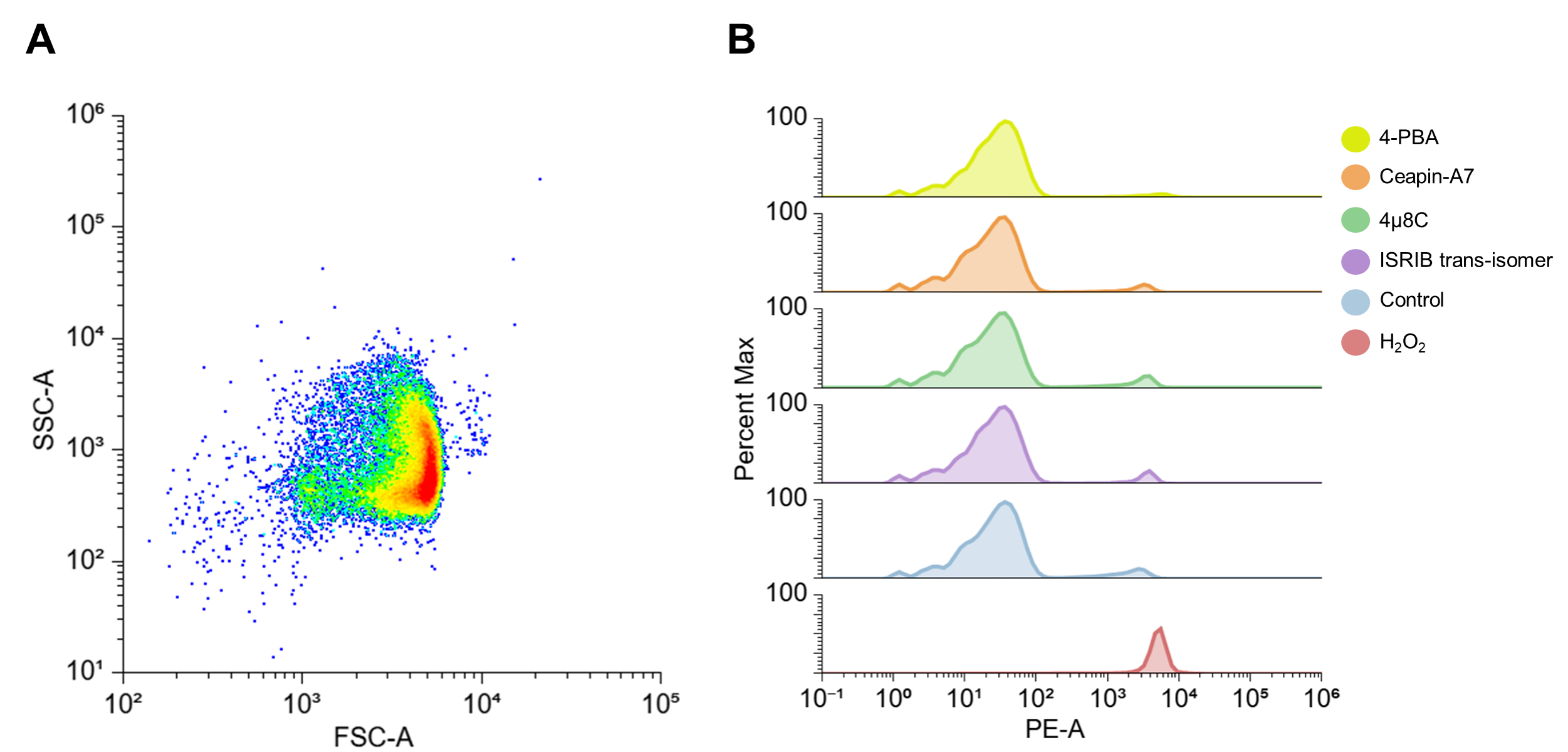


**Figure S2. Effect of ER stress and UPR^ER^ inhibitors on rainbow trout RBCs viability (****related to Fig. 9 and Fig. S3).** Cell viability was assessed by PI staining in RBCs exposed to 4-PBA (8 mM), Ceapin-A7 (12.5 µM), 4µ8C (10 µM), ISRIB trans-isomer (200 nM), untreated RBCs (negative control), and RBCs treated with H₂O₂ (positive control). (A) Representative forward scatter (FSC) vs. side scatter (SSC) dot plot of rainbow trout RBCs. (B) Representative PE fluorescence histograms of inhibitor-treated RBCs, control RBCs, and H₂O₂-treated RBCs. Flow cytometry data were processed using Floreada.io software.


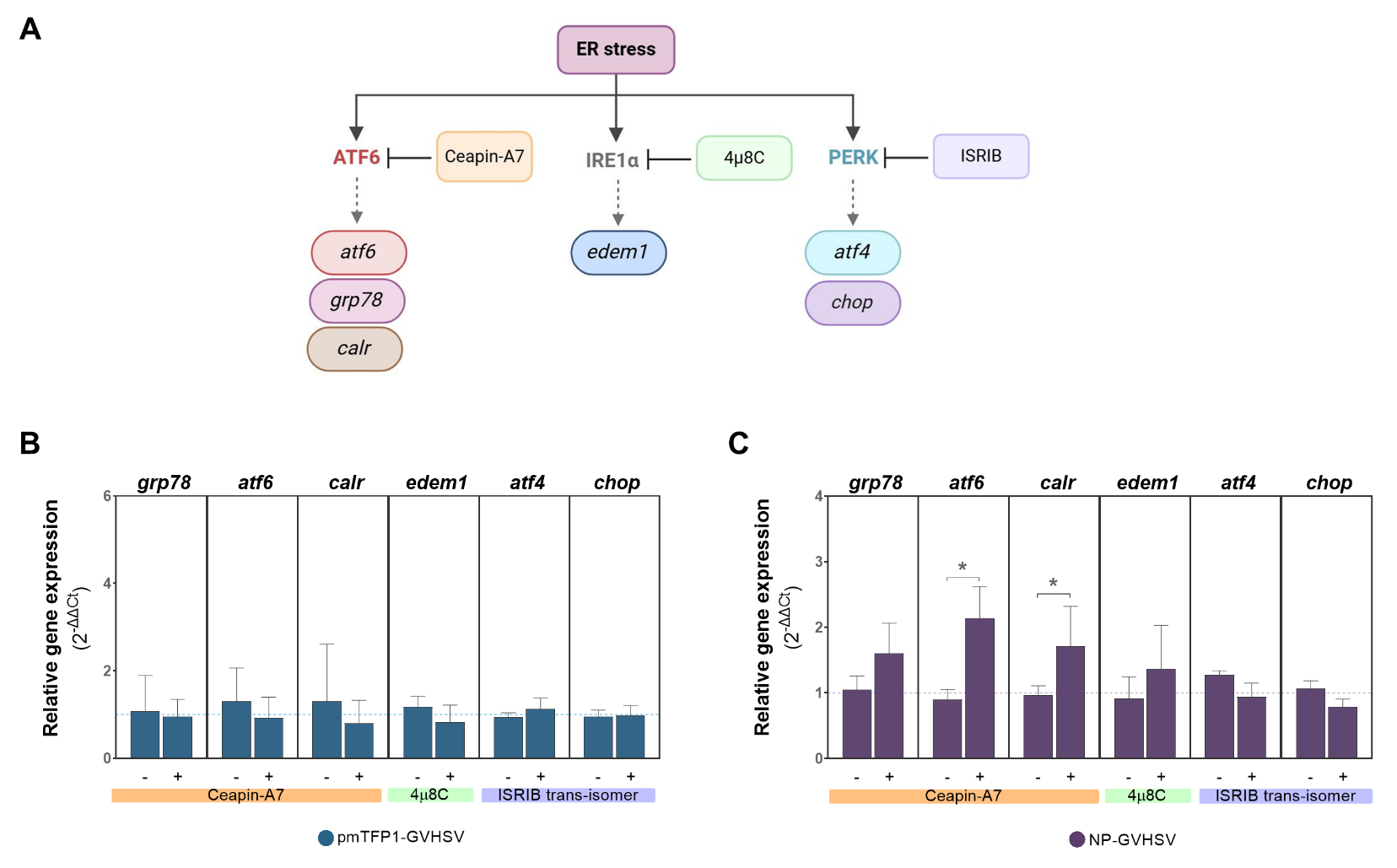
**Figure S3. Effect of ATF6, IRE1α, and PERK inhibition on UPR^ER^-related genes in rainbow trout RBCs in response to vaccine treatment (related to Fig. 9 and Fig. S2).** (A) Schematic representation of the three major UPR^ER^ branches: ATF6, IRE1α, and PERK, and their selective inhibitors: Ceapin-A7 (ATF6), 4u8C (IRE1α), and ISRIB trans-isomer (PERK). The target genes analyzed for each pathway are indicated. (B and C) Expression of *grp78*, *atf6*, *calr*, *edem1*, *atf4*, and *chop* in pmTFP1-GVHSV- or NP-GVHSV-treated RBCs upon Ceapin-A7 (12.5 µM), 4µ8C (10 µM), or ISRIB (200 nM) treatment. *ef1α* was used as the endogenous gene for normalization. Gene expression levels are represented as fold changes (2^-ΔΔCt^) relative to the control RBCs (treated with pmTFP1 or NP-iRFP, with or without inhibitors). Data are shown as mean ± standard deviation (n=3). Statistical analysis was performed by the Kruskal-Wallis test with Dunn's multiple comparison test. **P* <0.05 between treatments.


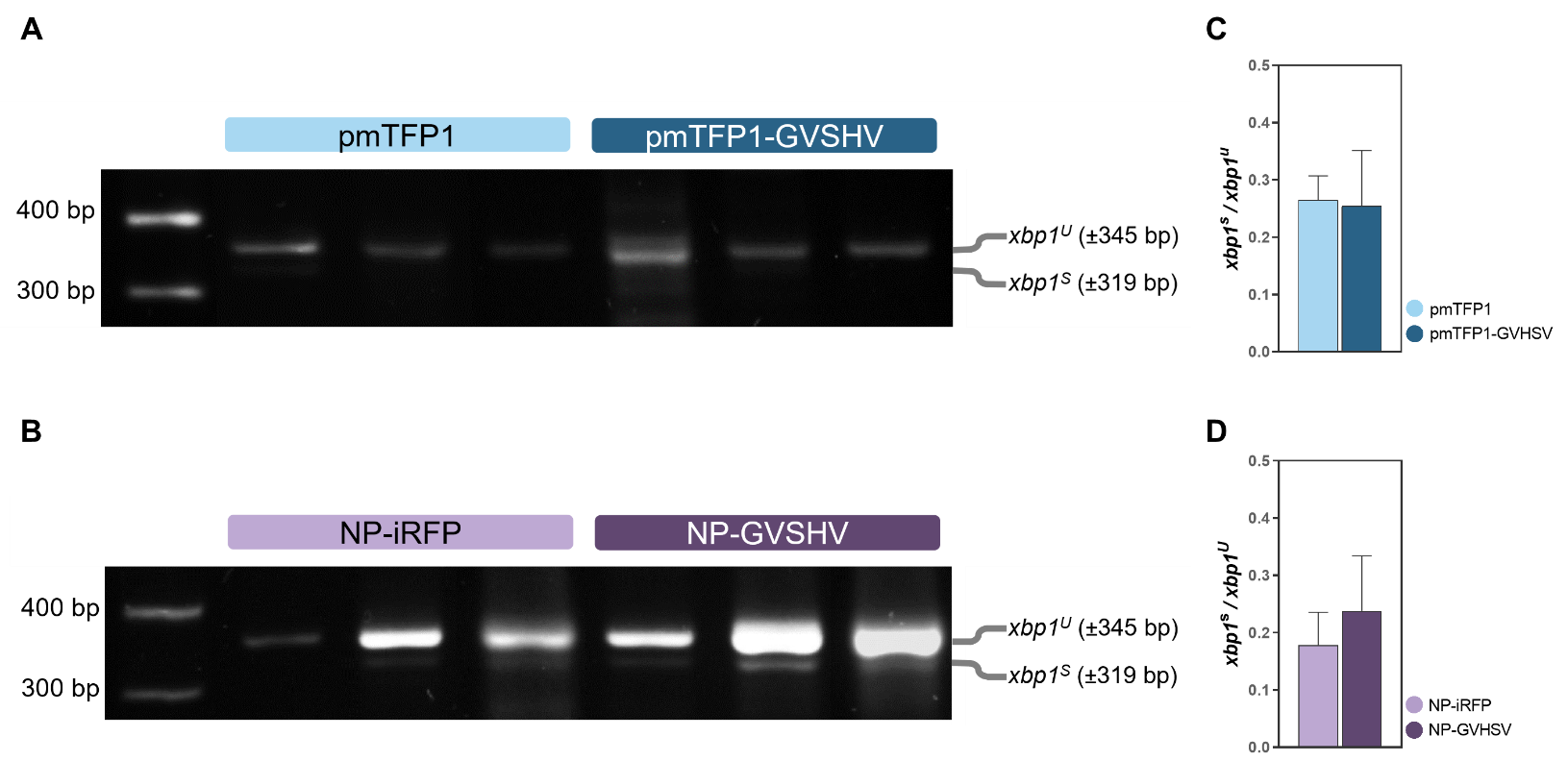
**Figure S4. Evaluation of *xbp1* mRNA splicing in RBCs from rainbow trout immunized with pmTFP1-GVHSV or NP-GVHSV (related to Fig. 10).** (A, B) Representative agarose gel of *xbp1* isoforms (*xbp1^U^* and *xbp1^S)^* of RBCs from individuals immunized with pmTFP1-GVHSV or NP-GVHSV, and their respective controls, pmTFP1 or NP-iRFP. Semi-qPCR products size corresponding to *xbp1^U^* and *xbp1^S^* mRNA and the DNA molecular weight marker are indicated in base pairs (bp). (C, D) Densitometric analysis of the semi-qPCR *xbp1* splicing assay in (A and B) was used to calculate the *xbp1^S^ / xbp1^U^* ratio.  Quantification of band densitometry was performed using Scion Image software. Plotted data represent mean ± standard deviation (n=3). The non-parametric Mann-Whitney test was used for statistical analysis between the two treatments.
